# The exon junction complex coordinates the cotranscriptional inclusion of blocks of neighboring exons

**DOI:** 10.1101/gad.353081.125

**Published:** 2026-01-01

**Authors:** Alexandra Bergfort, Jackson M. Gordon, Matthew R. Gazzara, Chuan-Tien Hung, Benhur Lee, Yoseph Barash, Karla M. Neugebauer

**Affiliations:** 1 Department of Molecular Biophysics and Biochemistry, Yale University, New Haven, Connecticut 06511, USA;; 2 Department of Genetics, University of Pennsylvania, Philadelphia, Pennsylvania 19104, USA;; 3 Department of Computer and Information Science, University of Pennsylvania, Philadelphia, Pennsylvania 19104, USA;; 4 Department of Microbiology, Icahn School of Medicine at Mount Sinai, New York, New York 10029, USA

**Keywords:** exon junction complex, pre-mRNA splicing, cotranscriptional splicing, long-read sequencing, coordinated splicing, splicing order

## Abstract

The exon junction complex (EJC) has roles in mRNA export and cytoplasmic quality control. However, the EJC is recruited to pre-mRNA by the spliceosome prior to the completion of splicing. When splicing is cotranscriptional, the EJC is deposited on nascent RNA early during synthesis, raising the question of whether the EJC regulates downstream RNA processing. Here we show, using long-read sequencing, that degron-mediated depletion of EJC component EIF4A3 leads to skipping of neighboring pairs of two or more exons on the same mRNA molecule. These data suggest that the entire “exon block” requires the EJC for inclusion. Introns flanking EJC-dependent exon blocks were longer and spliced after internal introns. In our working model, block exons are first spliced together to form a larger EJC-marked exon that promotes surrounding splicing events. Strikingly, analysis of 480 RNA binding protein knockdowns across two different human cell lines revealed block exons that are dependent on other splicing factors, indicating that coordinated splicing of adjacent exons is a general mechanism, of which the EJC is the dominant regulator. Cell type-specific coordinated splicing of adjacent exon pairs has been observed before. Here we identify the EJC as the main protein factor massively regulating this novel splicing mechanism in *trans*.

## Abstract (precis)

In this study, Bergfort et al. report that the exon junction complex (EJC) plays a major role in regulating cotranscriptional mRNA splicing of neighboring exons in *trans*. These exon blocks require the EJC for inclusion, which then promotes the coordinated splicing of adjacent exon pairs.

Most human genes contain multiple exons, and >90% can undergo alternative splicing, generating transcript isoforms with distinct functions (Wang et al. 2008). Current models of splicing mechanisms are heavily informed by in vitro studies and cryo-EM structures that use single-intron synthetic pre-mRNAs as substrates to reconstitute the splicing reaction (Rogalska et al. 2023; Shenasa and Bentley 2023). However, sequencing of chromatin- or Pol II-associated RNA libraries indicates that ∼75% of mammalian introns are removed cotranscriptionally (Tilgner et al. 2012; Nojima et al. 2015; Sousa-Luis et al. 2021; Zeng et al. 2022; Shine et al. 2024). A consequence of cotranscriptional splicing is that the pre-mRNA substrate is highly dynamic in vivo, and individual alternative splicing events may vary depending on preceding splicing reactions or the extent of RNA synthesis. Early splicing events can thereby recruit protein factors and also generate new *cis*-regulatory elements at exon–exon junctions, tuning the local chemical environment for nearby splicing reactions. Indeed, introns that are dependent on prior splicing events for their excision have already been reported (Kim et al. 2017). *Trans*-acting factors can bind the nascent RNA and generate a local protein environment that could directly influence nearby RNA processing steps. How the dynamic association of RNA binding proteins interacts with and regulates cotranscriptional splicing is largely unknown.

The exon junction complex (EJC) is assembled during splicing and binds mRNAs ∼24 nt upstream of exon–exon junctions (Le Hir et al. 2000; Schlautmann and Gehring 2020). We therefore reasoned that the EJC may help coordinate splicing from one intron to the next as the pre-mRNA is being synthesized. In mammals, the EJC is composed of EIF4A3, RBM8 (Y14), CASC3 (MLN51/Barentsz), and MAGOH. EIF4A3 is the main RNA binding component of the EJC, with ATPase activity that clamps the protein to RNA in a sequence-independent manner. The EJC has been extensively studied with respect to its involvement in mRNA export, translation, RNA surveillance via nonsense-mediated mRNA decay (NMD), *N*6-methyladenosine (m^6^A) deposition, and pre-mRNA splicing (Le Hir et al. 2016; Woodward et al. 2017; Schlautmann and Gehring 2020; Clyde 2023). For instance, siRNA knockdown of EJC subunits resulted in splicing alterations of ∼700 alternative and constitutive cassette exons. The majority of these exons showed increased skipping upon depletion of EJC components, suggesting that the EJC may be required for the activation of certain splicing events (Wang et al. 2014). EJC loading has also been reported to repress recursive splicing and cryptic splice site usage, suggesting that the EJC can influence proximal splice events (Blazquez et al. 2018; Boehm et al. 2018). Despite this wealth of information about the EJC, the detailed molecular basis for the effects of EJC depletion on nuclear RNA processing outcomes remains unclear.

To precisely define the role of EJC recruitment in cotranscriptional splicing, we used long- and short-read sequencing of both mature mRNA and chromatin-associated (nascent) RNA following degron-mediated EIF4A3 depletion. We found EIF4A3 to be critical for maintaining cotranscriptional splicing fidelity, with its depletion affecting splicing in 55% of genes with read coverage in our data sets, a substantially higher number than previously reported. Strikingly, we found that adjacent exon pairs were disproportionately skipped upon EIF4A3 depletion and that the EJC is critical for inclusion of these so-called “exon blocks.” Taken together, our study not only highlights a previously unknown role for the EJC in maintaining splicing fidelity but also reveals a novel mechanism whereby a *trans*-acting protein factor coordinates splicing across multiple exons and introns by generating a larger exon from smaller internal exons cotranscriptionally.

## Results

### EIF4A3 depletion causes skipping of multiple adjacent exons within genes

**Figure 1.**
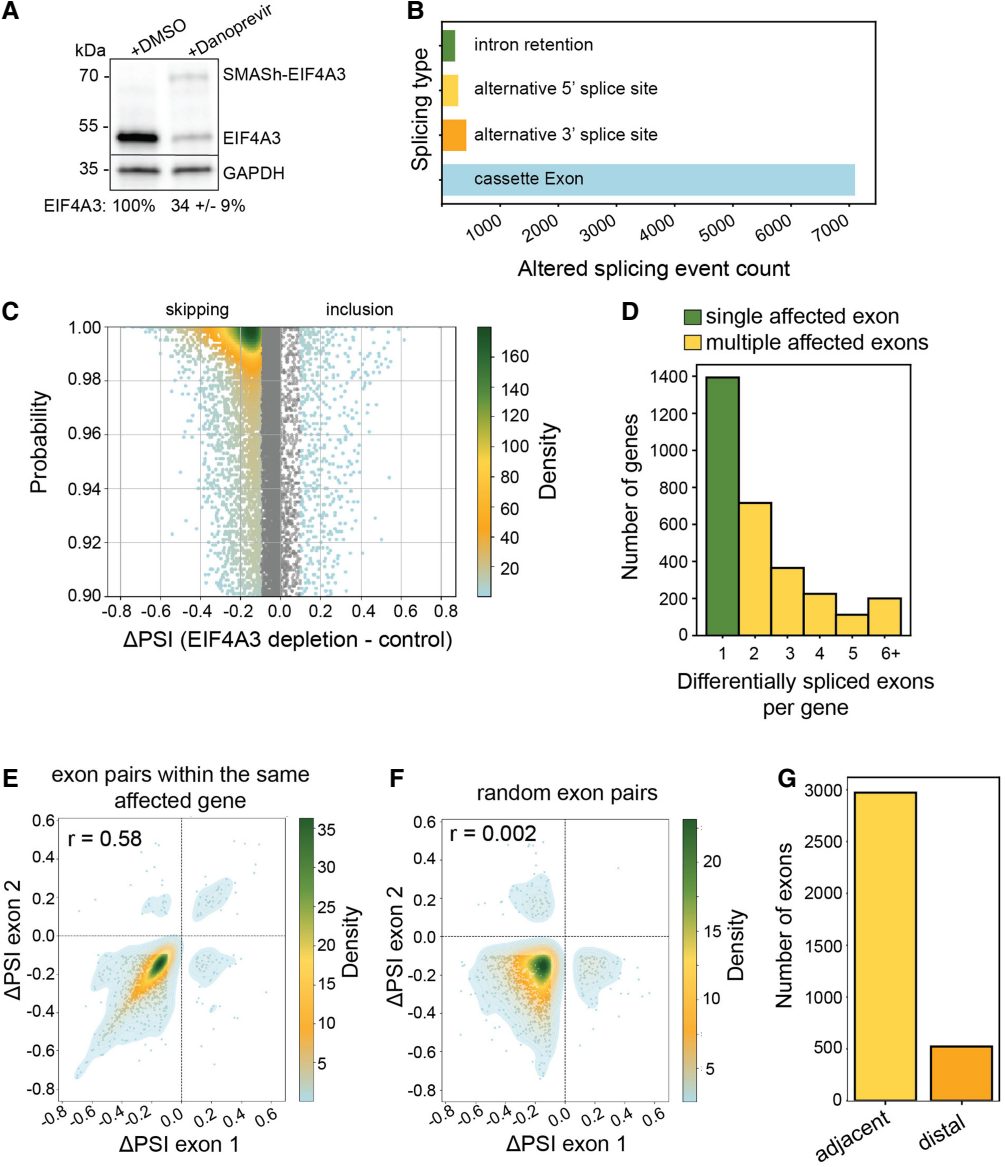
EIF4A3 depletion causes widespread skipping of neighboring exons. (*A*) Western blot showing EIF4A3 and GAPDH levels in SMASh-EIF4A3 HEK293T cells treated with DMSO or danoprevir for 24 h. (*B*) Number of altered splicing events by type after EIF4A3 depletion. (*C*) ΔPSI (*X*-axis) versus event probability (*Y*-axis; Whippet score) for cassette exons; color reflects local point density (2D Gaussian KDE). Threshold: probability ≥0.9 and ΔPSI ≥10% (mean of three biological replicates). (*D*) Histogram of differentially spliced exons per gene. (*E*) ΔPSI correlation of significantly altered exons from the same gene; the heat map shows point density. (*F*) Same as *E* but with randomly paired exons. (*G*) Bar chart showing relative positions of similarly affected exons (ΔPSI difference ≤0.02). (Yellow) adjacent, (orange) distal.

To investigate the role of the EJC in cotranscriptional splicing regulation, we focused on its core component EIF4A3 (Schlautmann and Gehring 2020). To achieve rapid depletion of EIF4A3 protein and to avoid off-target effects that are common with siRNA knockdowns, we used an N-terminal SMASh tag degron tag in HEK293T cells (Chung et al. 2015). In the absence of the drug danoprevir, the degron-attached self-cleaving protease effectively removes the tag from the protein so that the tag does not sterically interfere with the integrity of the protein complex (Supplemental Fig. S1A). The time required for depletion is highly dependent on the half-life of the untagged protein. Although EIF4A3 is relatively stable, we were able to achieve 66% depletion after 24 h of danoprevir treatment compared with DMSO-treated controls (Fig. 1A).

To first investigate how rapid EIF4A3 depletion affects pre-mRNA splicing, we analyzed poly(A)^+^ RNAs (mRNA) from three biological replicates with Illumina paired-end (short-read) sequencing, yielding a mean of 49 million uniquely mapped reads per sample (Supplemental Table S1). We quantified changes in alternative splicing, including intron retention, using the alternative splicing software suite Whippet (Sterne-Weiler et al. 2018). Extensive alterations in splicing were observed (7098 altered cassette exons), comparing EIF4A3 depletion versus control treatment (Fig. 1B). To ensure that the observed splicing changes resulted from EIF4A3 depletion and not danoprevir treatment, we treated wild-type HEK293T cells with danoprevir or DMSO. We observed relatively few changes in splicing (428 altered cassette exons), of which 53 (12.4%) overlapped with the changes observed for EIF4A3 depletion (Supplemental Fig. S1B). Thus, most observed splicing alterations resulted from EIF4A3 depletion rather than danoprevir alone.

Alternative splicing analysis revealed 7098 exons that were altered in their percent spliced in (PSI) by at least 10% with a Whippet probability score of at least 0.9 upon EIF4A3 depletion (Fig. 1B,C). Interestingly, 93% of exons displayed more frequent skipping compared with controls, and at least 59% of these exons were annotated as constitutively spliced exons (defined as being present in all annotated isoforms regardless of tissue or cell line). These results point toward a general role of EIF4A3 in maintaining splicing fidelity rather than regulating alternative splicing events per se. Notably, the 7098 differentially spliced exons originated from only 2971 unique genes, and more than half of these genes had two or more EIF4A3-dependent exons (Fig. 1D). A permutation test randomly distributing the number of observed splicing changes across the number of Whippet-quantified genes indicated higher than expected enrichment of altered splicing events per gene (Supplemental Fig. S1C). This prompted us to investigate whether significant exons within the same gene, referred to here as exon pairs, were skipped to roughly the same degree. Indeed, somewhat strong overall correlation of ΔPSI values between paired exons was observed (*r* = 0.58) (Fig. 1E), while a random pairing of all exons in the pool did not yield correlation (*r* = 0.02) (Fig. 1F). These results suggest that EIF4A3-dependent exon pairs may be regulated in a similar manner. We next wondered whether exon pairs were proximal or distal to each other within their parent gene. Relative position analysis of eIF4A3-regulated exons from the same gene that differed in ΔPSI by a maximum of 2% revealed that 85% of these exons are adjacent to each other (Fig. 1G). Taken together, these results suggest that EIF4A3 depletion often results in skipping of two or more neighboring exons.

### Depletion of other EJC components also leads to skipping of neighboring exons

**Figure 2.**
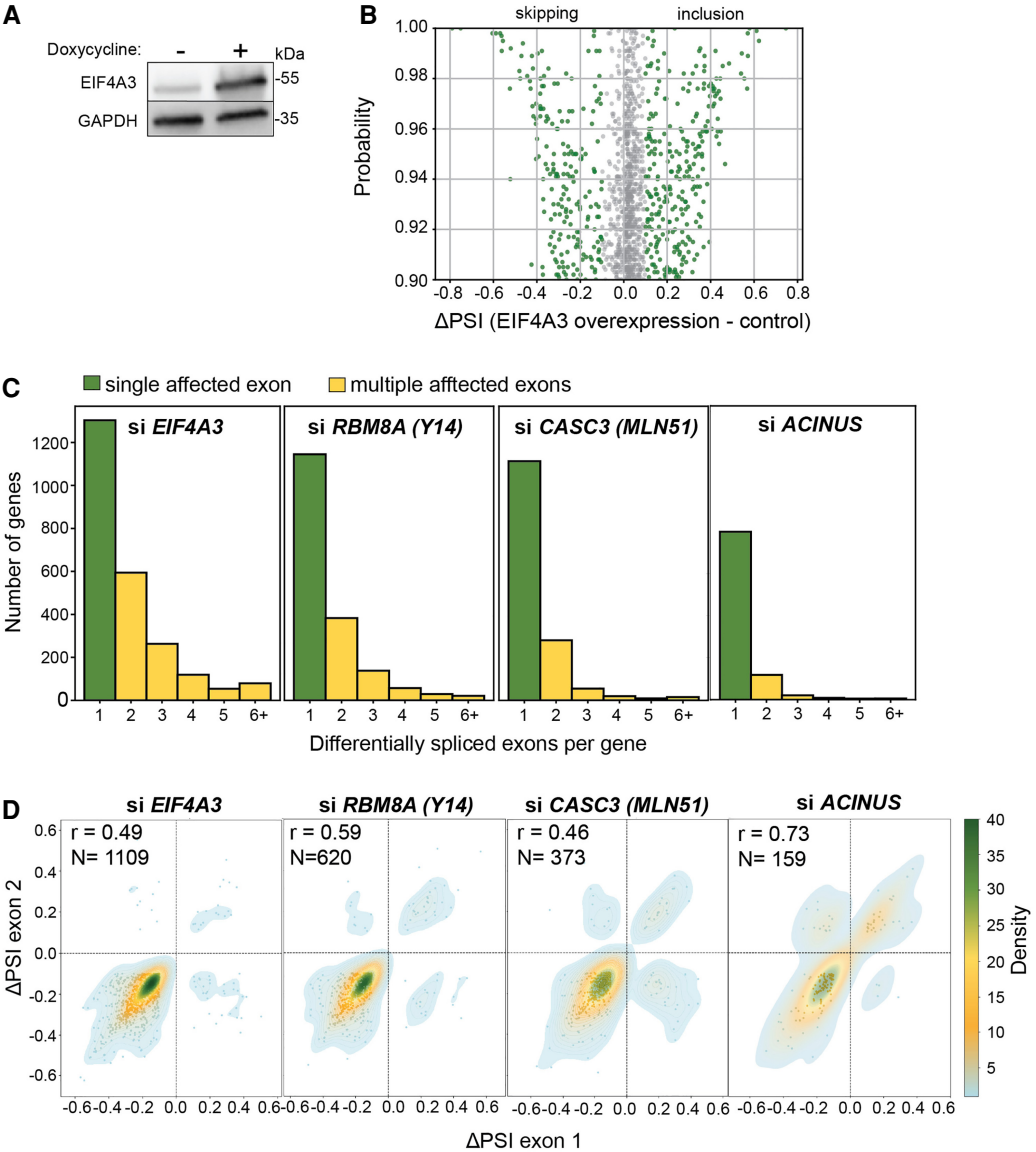
Coordinated inclusion of neighboring exons requires multiple EJC components. (*A*) Western blot showing EIF4A3 overexpression relative to GAPDH in HEK293 Flp-In T-REx cells after induction with doxycycline for 48 h. (*B*) ΔPSI (*X*-axis) versus event probability (*Y*-axis; Whippet score) for cassette exons upon EIF4A3 overexpression; color reflects local point density (2D Gaussian KDE). Threshold: probability ≥0.9 and ΔPSI ≥10% (mean of three replicates). (*C*) Histogram of differentially spliced exons per gene in siRNA knockdowns of EJC components in HeLa cells. (*D*) ΔPSI correlation of significantly altered exons from the same gene; the heat map shows point density.

Because EIF4A3 depletion led to high levels of exon skipping, we wondered whether EIF4A3 overexpression would result in widespread alternative exon inclusion. To test this, we generated a HEK293 Flp-In T-REx cell line with EIF4A3 overexpression under control of a tetracycline repressor (Dox-on) (Fig. 2A). mRNA short-read sequencing yielded 35 million uniquely mapped reads per sample (Supplemental Table S1). Only 474 exons were differentially spliced in cells overexpressing EIF4A3, in contrast to 7098 exons that were significantly affected by EIF4A3 depletion (Fig. 2B). This mild effect upon overexpression suggests that EIF4A3 does not act broadly on splicing independent of the other EJC components. To investigate whether knockdown of other EJC components induces widespread exon skipping, we analyzed publicly available RNA-seq data of siRNA knockdowns of *RBM8*, *CASC3*, and *EIF4A3* in HeLa cells (Wang et al. 2014). We also included a HeLa cell siRNA knockdown of *ACINUS*, a peripheral EJC factor with reported function in splicing (Hayashi et al. 2014; Rodor et al. 2016). Splicing analysis with Whippet (Sterne-Weiler et al. 2018) revealed 4635 differentially spliced exons in the *EIF4A3* knockdown sample, with 44.2% of exons overlapping with differentially spliced exons that we found upon EIF4A3 depletion in HEK293T cells (Supplemental Fig. S1D). Like our mRNA short-read data set after EIF4A3 depletion, the published EJC component siRNA knockdowns also revealed a significant enrichment of multiple affected exons per gene (Fig. 2C; Supplemental Fig. S1E). Interestingly, this was the case not only for si*EIF4A3* but also for si*RBM8A, siCASC3*, and *siAcinus*, with the strongest enrichments for EIF4A3 and RBM8A. Likewise, ΔPSI values of exons differentially spliced within the same gene showed a degree of correlation similar to that of our EIF4A3 depletion (Fig. 2D). Together, these data suggest that coordinated skipping of neighboring exons is regulated by the EJC and not by EIF4A3 independently. This is in line with previous findings that splicing regulation through the EJC is dependent on the integrity of this complex and not mediated by individual components (Wang et al. 2014).

### Neighboring “block exons” are coordinately skipped upon EIF4A3 depletion

**Figure 3.**
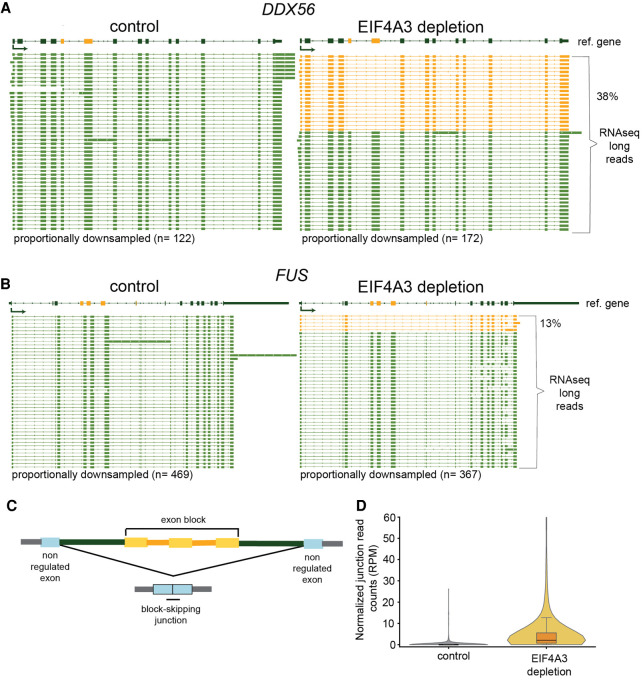
The EJC is required for inclusion of exon blocks at the single transcript level. (*A*) Genome browser shot of individual long reads of mRNAs mapped to the *DDX56* gene, showing control (*left*) and EIF4A3 depletion (*right*). Reference (ref.) gene is at the *top*, with coordinated block exons in orange. Reads are proportionally downsampled to 50 reads (total number of reads is indicated *below* the reads), with reads where exon blocks are skipped in orange and all other reads in green. The percentage of block-skipping reads is indicated at the *right* of reads. (*B*) Like *A* but for the *FUS* gene. (*C*) Schematic of block-skipping junctions, with the coordinated exon block in yellow (internal introns in orange) and surrounding exons in light blue. (*D*) Violin box plot illustrating the distribution of reads per million (RPM) spanning block-skipping junctions in control and EIF4A3 depletion conditions in mRNA long-read sequencing data sets (mean of three biological replicates). Values >60 were capped to avoid extreme outliers distorting the visualization.

To test whether skipping of EIF4A3-dependent exons with similar ΔPSI values was coordinated within the same molecule of mRNA, we used long-read sequencing of poly(A)^+^ RNA following 24 h EIF4A3 depletion in three biological replicates. Our data set contained on average 699,000 uniquely mapped long reads per sample, with a mean read length of 1708 nt (Supplemental Table S1). Mapped reads for individual examples displayed skipping of exon blocks in EIF4A3 depletion samples but not in control samples (Fig. 3A,B). These results were validated by RT-PCR for two individual genes: *FUS* and *DDX56* (Supplemental Fig. S2A,B). To further confirm EIF4A3 specificity, we performed a rescue experiment where EIF4A3 is transiently overexpressed during the degron-mediated depletion, followed by RT-PCR for *FUS* and *DDX56*. The rescue restored the wild-type (untreated) phenotype with respect to splicing, and the block exon-excluded isoforms are only visible in the EIF4A3 depletion (Supplemental Fig. S2C–E). This shows that the findings are specifically due to EIF4A3 depletion and not some other effect of, for example, danoprevir that was not detected in the sequencing control.

Is the inclusion or exclusion of block exons an example of alternative splicing? Based on short-read sequencing of mRNA, we identified 3105 exons in coordinated exon blocks (that have an adjacent exon with a ΔPSI difference of maximal 0.02). These exons form 1060 blocks over 1016 genes, because some genes contained multiple exon blocks; 75.3% of blocks are formed by two exons, while the remaining 24.7% of blocks have a median of three exons (mean = 3.9 exons). The mean ΔPSI of these exons is 19.8%, and at least 65% of these exons are constitutive (defined here as present in all annotated RNA isoforms), compared with 54% of constitutive exons among the single EIF4A3-regulated exons (EIF4A3-regulated exons that are not in blocks) (Supplemental Fig. S2F). These data reinforce our view that the requirement of the EJC for block exon inclusion does not reflect a function in canonical alternative splicing even though alternative exons may be included within the identified blocks.

To further analyze the effect of block exon exclusion on the mRNAs detected upon EIF4A3 depletion, we defined a feature called “block-skipping junction” (see Fig. 3C), representing detected exon–exon junctions formed when exons upstream of and downstream from a block are spliced together. Using the coordinates of block-skipping junctions extracted from the mRNA short-read data set, we searched the mapped long-read sequencing mRNA reads for deletions of the exon block region based on annotations in the compact idiosyncratic gapped alignment report (CIGAR string) for each read (Fig. 3C). We detected reads over 58.5% of block-skipping junctions (Fig. 3D), with a mean of 12.5% reads covering the respective region carrying the block-skipping junction. The remaining 43% of block-skipping junctions remained undetected, likely due to the lower sequencing depth afforded by long- versus short-read sequencing. The majority of block-skipping junctions were only observed upon EIF4A3 depletion, in line with the previous observations that at least 65% of block exons are constitutive exons. Taken together, our results suggest that EIF4A3 acts as a splicing fidelity factor to ensure coordinated inclusion of multiple neighboring exons that make up exon blocks.

The EJC plays a critical role in RNA surveillance through the NMD pathway, whereby a premature termination codon upstream of an EJC is sufficient to recruit UPF1 and mark the RNA for degradation. Because the EJC is necessary for NMD, we reasoned that the observed increase in transcripts with exon block skipping may be due to reduced NMD activity in the absence of EIF4A3. To test this hypothesis, we first carried out differential gene expression analysis using DESeq2 (Love et al. 2014). While danoprevir treatment of wild-type HEK293T cells had no significant effect on gene expression, we identified 1820 genes with significantly altered expression upon EIF4A3 depletion (at least twofold increase or decrease with an adjusted *P*-value of ≤0.05) (Supplemental Fig. S3A). However, only 184 differentially expressed genes overlap with the altered cassette exon genes detected by Whippet, and only 84 genes are also identified as coordinated exon block carriers (Supplemental Fig. S3B). Thus, the emergence of transcript isoforms with skipped exon blocks due to stabilized NMD could only account for 8% of the 1016 identified exon block genes. In addition, 47.7% of skipped blocks are frame-preserving (Supplemental Fig. S3C). Thus, while we cannot completely rule out the possibility of NMD involvement in stabilizing block-skipped transcripts, our data suggest that the appearance of block-skipping transcripts upon EIF4A3 depletion cannot be fully explained by reduction in NMD activity.

### EJC-dependent exon blocks are spliced cotranscriptionally

**Figure 4.**
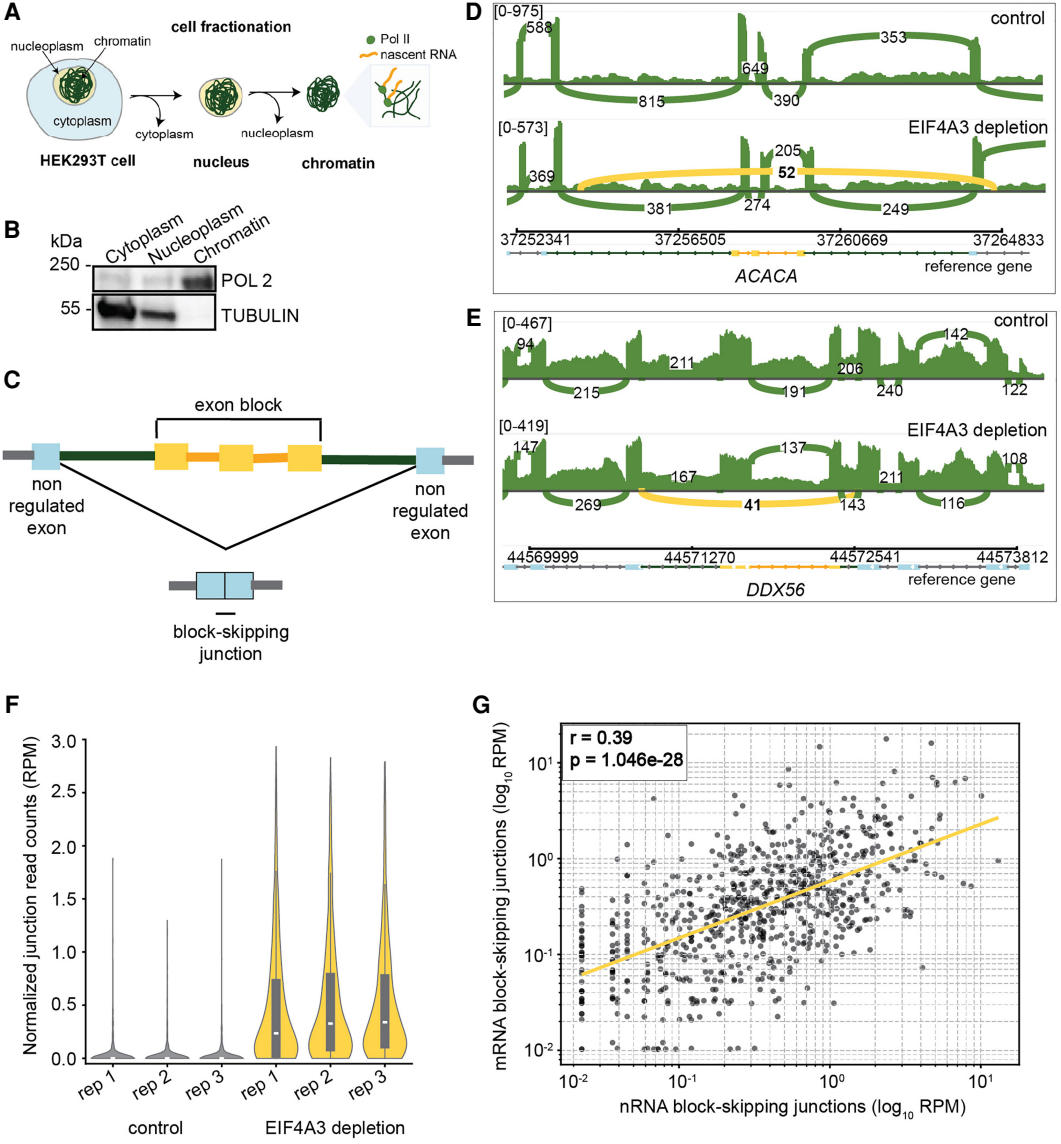
EJC-dependent block exon inclusion occurs cotranscriptionally. (*A*) Illustration of the cell fractionation steps yielding nascent RNA (nRNA). (*B*) Western blot showing a successful cell fractionation, with tubulin as a cytoplasmic marker and RNA polymerase II (Pol 2) as a chromatin marker. (*C*) Schematic of block-skipping junctions. (*D*) Sashimi plot of nRNA short reads (green) aligned to the *ACACA* gene. The block exon region in yellow–orange is indicated in the reference gene *below*. Block-skipping junction read coverage is highlighted in yellow. (*E*) Like *D* but for the *DDX56* gene. (*F*) Violin plot showing normalized read count in reads per million (RPM) in nRNA short reads spanning block-skipping junctions in control and EIF4A3 depletion conditions for three biological replicates (rep). (*G*) Scatter plot showing correlation of RPM for block exon-skipping junctions in mRNA versus nRNA short-read sequencing data sets. Pearson correlation and *P*-value are indicated in the *top left* of the plot.

The EJC is deposited on pre-mRNAs during the splicing reaction, which has been shown to occur mostly cotranscriptionally in mammals (Shine et al. 2024). We therefore hypothesized that decoration of nascent RNA with EJCs would affect neighboring cotranscriptional splicing reactions. To test whether EIF4A3-dependent block exon inclusion occurs cotranscriptionally or post-transcriptionally, we fractionated the SMASh-EIF4A3 HEK293T cells following 24 h EIF4A3 depletion and extracted nascent RNA (nRNA) from the chromatin fraction (Fig. 4A,B). Illumina paired-end short-read sequencing of nRNA yielded a mean of 91 million uniquely mapped reads per sample for three biological replicates of EIF4A3 depletion and control, respectively (Supplemental Table S1).

If EIF4A3-dependent block exon inclusion occurs cotranscriptionally, we would expect to see block exon-skipping junctions (junction between upstream of and downstream from block exons) in nascent RNAs (Fig. 4C). Furthermore, we would expect to see this junction mainly in the EIF4A3 condition, because most block exons are constitutive. Sashimi plots of individual genes indeed indicated block-skipping junction reads only upon EIF4A3 depletion (Fig. 4D,E). Furthermore, we analyzed read coverage over exon–exon junctions in the entire nRNA data set and detected 75.6% of block exon-skipping junctions previously defined by the mRNA short-read data. Nearly all these junctions were only detected in the EIF4A3 depletion condition (Fig. 4F). Normalized read counts (reads per million [RPM]) slightly correlate for block exon-skipping junctions in mRNA and nRNA (Fig. 4G). Taken together, these data indicate that EIF4A3 promotes cotranscriptional inclusion of exon blocks.

**Figure 5.**
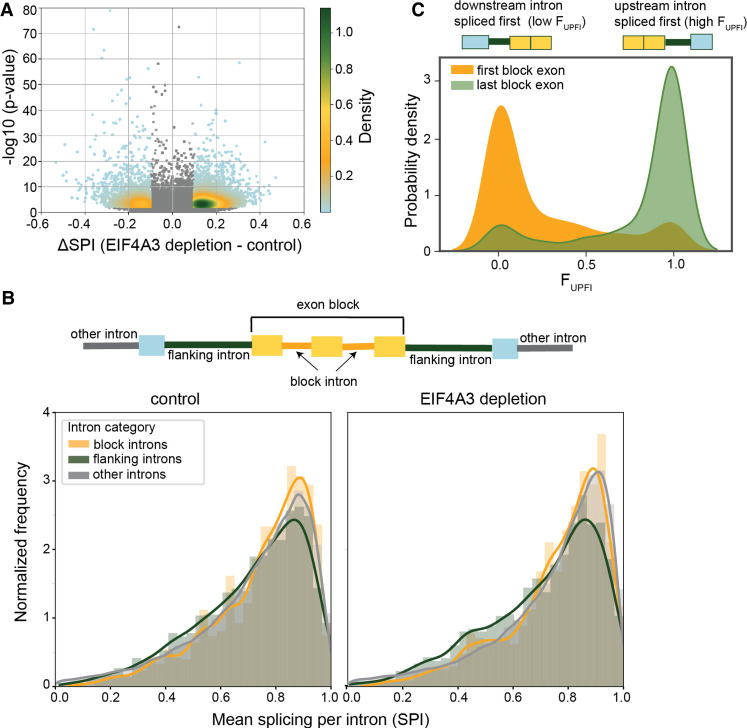
Introns within exon blocks are spliced out before the whole block is spliced in. (*A*) ΔSPI (*X*-axis) versus *P*-value (*Y*-axis); color reflects local point density (2D Gaussian KDE). Threshold: *P*-value ≤0.01 and ΔSPI ≥10% (mean of three replicates). (*B*) Normalized frequency histogram of SPI values for block internal introns (orange), block-flanking introns (dark green), and other introns (gray) in control (*left*) and EIF4A3 depletion (*right*) conditions. (*C*) Splicing order of introns surrounding the first (orange) and last (green) exons of coordinated exon blocks in control cells. The *Y*-axis is probability density (kernel density estimate), and the *X*-axis is fraction of upstream intron spliced first (*F*_UPFI_).

Because EIF4A3 depletion caused widespread exon skipping, we wondered whether intron removal efficiency of surrounding introns was affected as well. To assess cotranscriptional splicing efficiency of introns in the context of EIF4A3 depletion, we used SPLICE-q (de Melo Costa et al. 2021) to calculate splicing per intron (SPI) values from our short-read sequencing of the nRNA data set. Between control and EIF4A3 depletion, we identified 25,209 introns with significantly (*P*-value ≤0.01, ΔSPI minimum 10%) altered SPI, with 69.7% of these introns being more efficiently spliced upon EIF4A3 depletion (Fig. 5A). Introns with altered SPI originated from 4223 genes, meaning that 40.9% of detected genes (10,323) were altered in splicing; 62.5% of block exon genes and 44.5% of single EIF4A3-regulated exon genes overlapped with genes in which intron splicing was affected by EIF4A3 depletion, suggesting that altered intron splicing efficiencies may impact exon skipping (Supplemental Fig. S4A).

Interestingly, independent of EIF4A3, introns flanking coordinated exon blocks were spliced less efficiently than other introns, and block internal introns are spliced more efficiently, suggesting that block internal introns may be spliced before other introns in the same transcript (Fig. 5B). To further test this hypothesis, we used Insplico (Gohr et al. 2023), which estimates the order of intron removal with respect to a given exon. We first analyzed our short-read nRNA control. In agreement with our previous results, first exons of blocks displayed a fraction of upstream flanking intron spliced first (*F*_UPFI_) of 0, meaning that the block internal intron is spliced first (Fig. 5C). Likewise, most last block exons had a *F*_UPFI_ of 1, meaning again that the upstream block internal intron was spliced first (i.e., before the downstream flanking intron) (see Fig. 5B). Insplico yielded almost identical results for the EIF4A3 depletion data set, indicating that the order of intron removal is EIF4A3-independent (Supplemental Fig. S4B). Taken together, these data suggest that introns are generally removed more efficiently in the absence of EIF4A3. However, introns that flank exon blocks are spliced with low efficiency independent of EIF4A3 and may be spliced after exon block internal introns.

### Coordinated exon blocks are surrounded by weak splice sites and long introns

**Figure 6.**
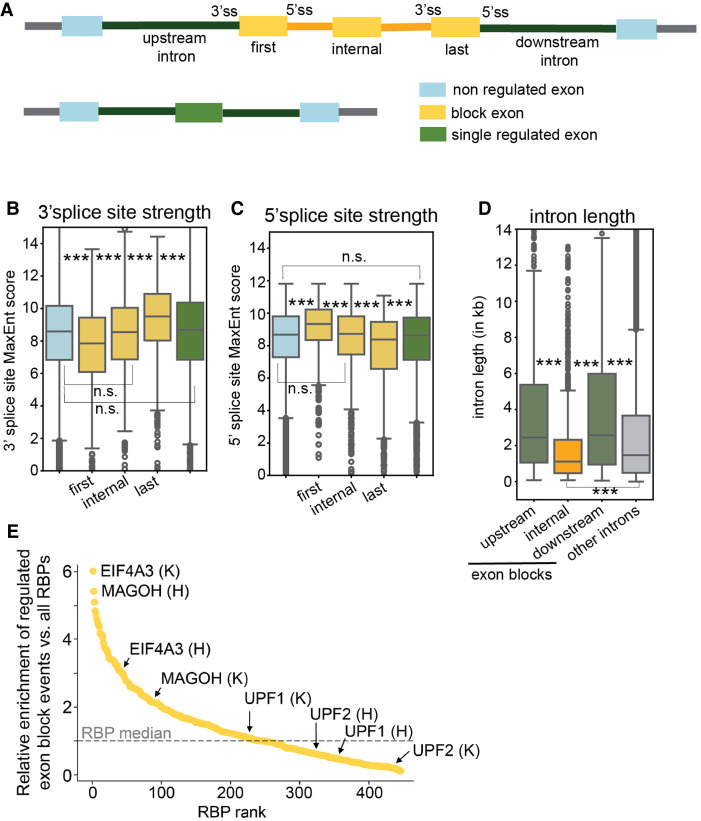
Coordinated exon blocks are surrounded by weak splice sites and long introns. (*A*) Color scheme for *B*–*D*. (*B*) Box plot of 3′ splice site strength of different exon categories, with MaxEnt score (Yeo and Burge 2004) on the *Y*-axis. (***) *P* < 0.001, (n.s.) not significant. (*C*) Like *B* but for 5′ splice site strength. (*D*) Intron length (in kilobases) of exon block internal (orange) and surrounding (dark green) introns compared with all other introns of genes expressed in the data set (gray). Significance is like in *B*. (*E*) Relative enrichment of exon block events per RBP (fraction of RBP-regulated AS events that are exon blocks compared with the median of all RBPs) for >480 RBP knockdown experiments from ENCODE HepG2 and K562 cells using MAJIQ v2 (see the Materials and Methods). EJC components in both cell types (HepG2 [H] and K562 [K]) are highlighted, as well as the NMD factors UPF1 and UPF2. The dashed line represents the median (relative enrichment = 1).

To further examine exon blocks that are spliced in a coordinated fashion, we analyzed the splice site strength of these exons (Fig. 6A), separating first, internal, and last exons of coordinated exon blocks and then calculating MaxEnt scores (Yeo and Burge 2004). While single EIF4A3-regulated exons did not have significantly different splice site strengths compared with non-EIF4A3-regulated exons, first exons of a block have significantly weaker and last exons of a block have significantly stronger 3′ splice sites, which is also visible in a distinct logo for the 3′ splice site of the first block exon (Fig. 6B; Supplemental Fig. S5A). The opposite is the case for 5′ splice sites. The MaxEnt scores indicate significantly weaker 5′ splice sites for this exon category and significantly stronger 5′ splice sites for first block exons (Fig. 6C), even though the 5′ splice sites of block exons displayed only small differences at positions −3 and +3 (Supplemental Fig. S5B). This indicates that, overall, collectively spliced exon blocks are surrounded by weaker splice sites and have stronger splice sites facing the internal introns.

Further analysis of block internal introns shows that they are significantly shorter, while the encompassing introns are significantly longer than other introns (compared with all expressed genes) (Fig. 6D). We found exon length to be slightly but significantly longer for block exons compared with other exons and single EIF4A3-regulated exons to be significantly shorter (Supplemental Fig. S5C). In accordance with the long surrounding introns, block exon genes are significantly longer than other genes, including genes with single EIF4A3-regulated exons (Supplemental Fig. S5D). Gene ontology analysis for biological processes yielded an only slight enrichment of processes related to cell division for both Whippet-detected differentially spliced genes and block exon genes, suggesting that EIF4A3-regulated splicing is not specific for a certain gene function (Supplemental Fig. S6). Taken together, these analyses show that EIF4A3 regulates coordinated splicing of exons in blocks that are flanked by weak splice sites and long introns, while block internal splice sites are stronger.

To investigate whether block exon inclusion could be regulated through an RNA binding protein (RBP) motif that, under control conditions, would be covered by EIF4A3, we analyzed a 10 nt region around the canonical EJC binding site (24 nt upstream of the exon–exon junction) within the first block exon. RBPmap (Paz et al. 2014) detected motifs for 132 RBPs that could potentially be obstructed by EIF4A3. GO term analysis showed 69-fold enrichment of the biological process “regulation of alternative mRNA splicing, via spliceosome.” However, a similar result was obtained when carrying out the analysis with the same number of randomly selected exons that are not EIF4A3-regulated cassette exons. Similarly, ESEfinder (Cartegni et al. 2003) yielded a comparable enrichment of SR protein and hnRNP protein binding sites for first block exons and control exons. Analysis of block exon sequences using MEME software (Bailey and Elkan 1994; Bailey et al. 2015) did not yield any significant motif enrichment over non-EIF4A3-regulated exons.

As an alternative to RBP motif analysis, we looked for block exon skipping in RNA-seq experiments following many RBP depletions from the ENCODE consortium (Van Nostrand et al. 2020). We analyzed a large set of >480 short-read RNA-seq experiments from shRNA depletion of various RBPs for splicing changes across two cell types (245 RBP knockdowns in HepG2 and 244 RBP knockdowns in K562) using MAJIQ v2 (Vaquero-Garcia et al. 2016; Vaquero-Garcia et al. 2023). Using MAJIQ v2 coupled with MOCCASIN (Slaff et al. 2021) allowed us to detect both annotated and unannotated splice junctions associated with block exon skipping while correcting for known and unknown confounders. Such confounder corrections are not offered by Whippet but are critical in these data due to strong batch effects (Van Nostrand et al. 2020). In addition, the MAJIQ v2 modulizer allows for automated detection of block exon splicing as a subtype, similar to the definitions described above (see the Materials and Methods).

We assessed which RBP knockdown experiments affected the largest fraction of block exon event types compared with all other alternative splicing event types defined by MAJIQ v2 (e.g., cassette exons, intron retention, alternative splice sites, etc.). Strikingly, knockdown of EIF4A3 and knockdown of MAGOH showed the largest fraction of block exon events in K562 cells and HepG2 cells, respectively (Fig. 6E; see Supplemental Table S3 for all counts). We compared splicing changes detected in our HEK293T EIF4A3 depletion data set with the ENCODE-derived EJC data and the EJC siRNA knockdowns in HeLa cells (Supplemental Table S4). The overlap between our HEK293T data and the previously published HeLa, HepG2, and K562 cell data is highly significant, with high correlation of dPSI values for overlapping regulated splice events. Taken together, our findings suggest that EJC components are regulators of block exon usage in multiple cell types and regulate a proportionally larger amount of these types of events relative to hundreds of other RBPs analyzed.

Previous studies suggested a role of the EJC in recursive splicing (Blazquez et al. 2018; Boehm et al. 2018). We therefore analyzed the capacity of block exons to undergo recursive splicing by determining the MaxEnt score (Yeo and Burge 2004) for a recursive 5′ splice site (Supplemental Fig. S7); 5.2% of coordinated block exons have a potential recursive 5′ splice site with a MaxEnt score >5.52 (a threshold that detects 90% of human canonical 5′ splice sites) (Blazquez et al. 2018), compared with 2.9% of non-EIF4A3-regulated exons. This indicates that only a slight enrichment of exons that could potentially undergo recursive splicing is found in exons that are coordinatively skipped upon EIF4A3 depletion. However, no significant difference in recursive splice site strength could be detected for block exons versus nonregulated exons. In contrast and in line with previous studies, exons that could potentially undergo recursive splicing were enriched among the single EIF4A3-regulated exons (16.1% above threshold). Additionally, those exons display significantly stronger recursive 5′ splice sites compared with block exons and nonregulated exons. Our data suggest that EIF4A3 regulates single exon splicing and collective exon inclusion via different mechanisms. Single regulated exons are enriched for alternative exons and might be regulated by EIF4A3 through the repression of recursive splicing, as described previously (Blazquez et al. 2018; Boehm et al. 2018). Coordinated exon blocks, on the other hand, appear to be dependent on the EJC and/or other *trans*-acting factors for the recognition of flanking splice sites, such that the splicing order is crucial for inclusion of these mostly constitutive exons.

## Discussion

A variety of coordinated pre-mRNA processing events, including transcription start site coupling to polyadenylation sites and coupling of exon pairs, have been identified and are the subjects of active investigation in the field (Tilgner et al. 2015, 2018; Anvar et al. 2018; Fiszbein et al. 2019; Hardwick et al. 2022; Alfonso-Gonzalez et al. 2023; Calvo-Roitberg et al. 2025). Although a role for one or more RBPs in these activities is often envisioned, no RBP has been specifically implicated in coordinated splicing events. Here we leveraged long- and short-read sequencing of mRNA and nascent RNA after acute EIF4A3 depletion to investigate the potential for the EJC to act in splicing coordination in human cells. This phenomenon was previously noticed during analysis of short-read sequencing of untreated human samples and referred to as “tandem exon inclusion” based on inference from Quesnel-Vallierès et al. (2024). Likewise, using single-nucleus isoform RNA sequencing, cell type-specific inclusion of adjacent exon pairs was reported in human brain tissues (Hardwick et al. 2022). By showing that EIF4A3 depletion drastically affects cotranscriptional splicing in a way that produces stable aberrant mRNAs that lacked several exons in a row, we have revealed that the EJC is the dominant regulator of the collective inclusion of two or more neighboring exons.

**Figure 7.**
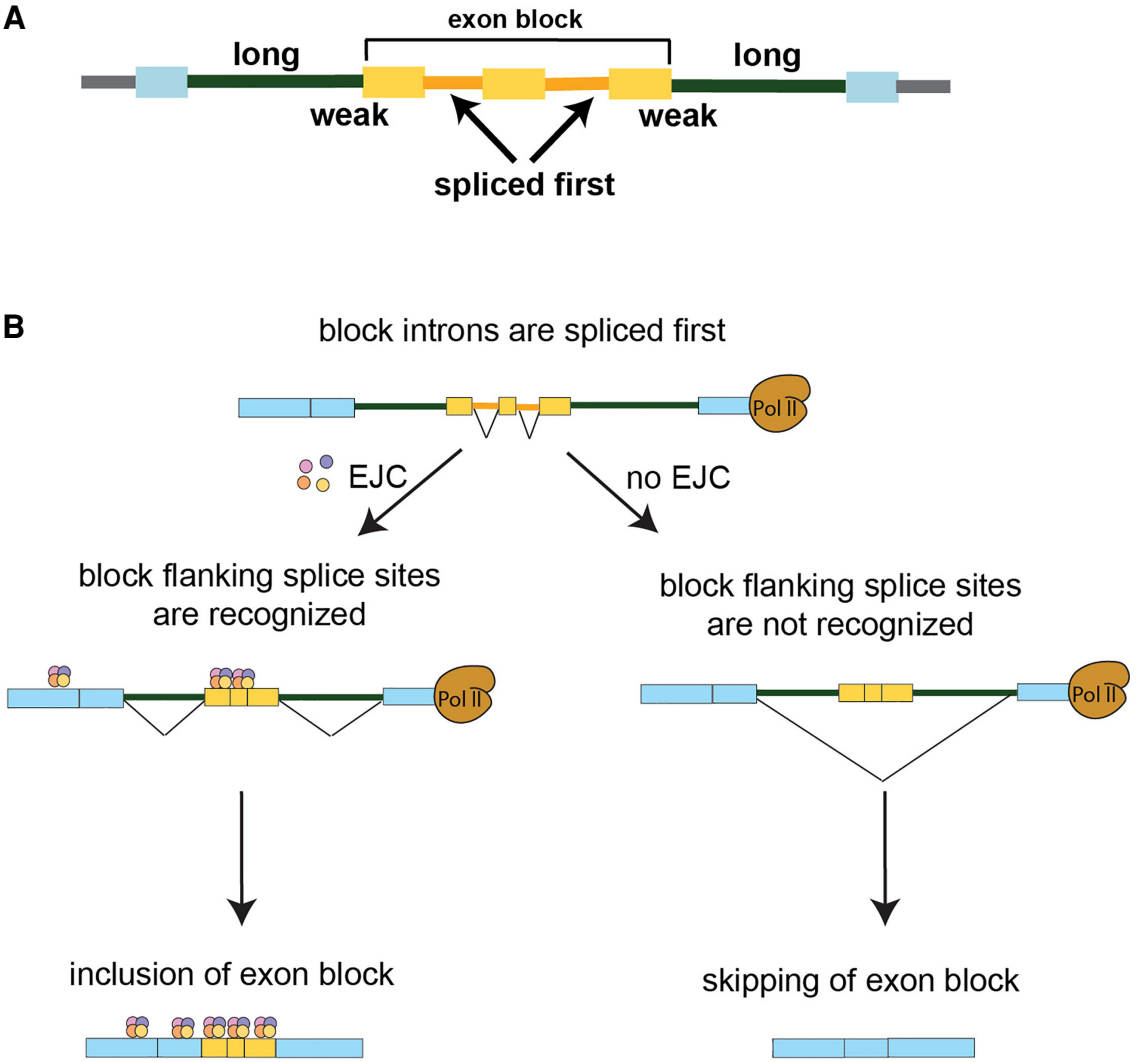
Working model of EJC-mediated inclusion of exon blocks. (*A*) Schematic model of an exon block-containing gene. Exon blocks are flanked by weak splice sites and long introns. Block internal introns are spliced first. (*B*) Model of EJC-mediated block exon inclusion. (*Left* panel) In the first step, block internal introns are spliced, leading to deposition of the EJC upstream of splice junctions. The EJC helps recognition of the weak flanking splice sites, facilitating inclusion of the entire exon block. (*Right* panel) In the absence of the EJC, the exon block is not recognized as exonic region and gets excised from the pre-mRNA as part of the intron.

In this study, we identified >1000 blocks of coordinated exons—either included or excluded—in individual mRNA transcripts. These blocks of coordinately spliced exons are flanked by weak splice sites and long introns that are spliced later than block internal introns. Our work suggests a model whereby the splicing of block internal introns leads to the deposition of the EJC on the exon block first, in turn promoting recognition of these combined exons by the spliceosome (see Fig. 7). This step implies that an exon definition mechanism may be involved, whereby the 3′ and 5′ splice sites are recognized once the exon is bound by *trans*-acting factors (such as the EJC and/or other RNA binding proteins like SR proteins) that promote U2 and U1 snRNP recruitment, respectively (Berget 1995; Rogalska et al. 2023). The expectations of this possible mechanism are consistent with the observation that the EJC binds nearly all internal exons (Morillo et al. 2023), because EJCs and other factors will accumulate on the block exons once they are spliced together. In the absence of the EJC, the spliceosome would not recognize the block exons, and the entire set of exons within the block would be skipped. This model is also supported by our analysis showing that depletion of other RBPs (such as U2AF1, HNRNPC, and HNRNPK) also yields alterations in coordinated splicing of exons in a block (Fig. 6E; Supplemental Table S3). Strikingly, the depletion of EJC components EIF4A3 and MAGOH had the most robust effect on block exon exclusion, suggesting that the EJC is the main determinant of this splicing behavior in humans. Taken together, our model describes a widespread mechanism of cotranscriptional splicing coordination, whereby the recruitment of *trans*-acting factors from the first set of splicing reactions is necessary to stimulate subsequent, neighboring splicing events.

Previous studies have reported that the order of intron excision does not always correlate with the order of intron synthesis (Tilgner et al. 2015; Kim et al. 2017; Herzel et al. 2018; Drexler et al. 2020; Marasco and Kornblihtt 2023). Moreover, some genes are prone to a specific order of excision for each intron. This finding is in line with our observation that exon block internal introns are spliced before the upstream flanking intron is removed. Work from Drexler et al. (2020) suggests that a combination of *cis*-regulatory elements including intron length, exon length, and splice site sequences contributes to splicing order decisions. Indeed, we found that the same *cis*-acting elements correlate with a fixed splicing order within and surrounding EIF4A3-regulated block exons, independent of EIF4A3. Other investigators showed that the splicing order is a strict requirement in certain genes (Kim et al. 2017), while some genes show transcript-specific “all spliced” or “all unspliced” behavior for certain genes (Reimer et al. 2021). The mechanisms underlying these phenomena remain unclear. Our study provides the first insights into at least one mechanism that governs splicing order. For EIF4A3-regulated exon blocks, the splicing order is indispensable to allow for inclusion of these exons. Interestingly, while delayed intron splicing (which we observed for upstream block introns) was reported to be mostly associated with alternative exons (Kim et al. 2017), the majority of exons that we identified as spliced in blocks are constitutive. Thus, splicing order is crucial for the regulation of not only alternative but also constitutive splicing.

Despite extensive research aimed at uncovering the functions of the EJC, the role of this complex in the nucleus and in splicing was unclear. The EJC is deposited on exon–exon junctions during splicing, with EIF4A3 first recruited to the spliceosome via CWC22 (Barbosa et al. 2012; Steckelberg et al. 2012). Our findings are consistent with the notion that nascent RNAs are decorated with EJCs cotranscriptionally, when many splicing events take place. While previous studies found that the EJC acts as a suppressor of back-splicing by sterically hindering the use of recursive splice sites (Blazquez et al. 2018; Boehm et al. 2018), the activity that we identified implies that the EJC additionally acts as a feed-forward splicing factor, promoting subsequent splicing reactions after it has been loaded.

Future work will be required to uncover more fine-grained features of block exons and whether block exons are regulated by other *trans*-acting factors in a tissue-specific manner. This will be important in understanding splicing variations that happen in healthy and diseased human tissues. Indeed, previous analysis of alternative splicing modules defined by MAJIQ over representative tissues from GTEx shows that in any given single tissue, between 8.6% and 13.8% of all modules display block exon-like behavior with a minimal usage of the block-skipping junction of at least 5% (Vaquero-Garcia et al. 2023). Approximately one-third of all known disease-causing mutations are predicted to cause aberrations in pre-mRNA splicing (Lim et al. 2011; Rogalska et al. 2023). Understanding the fundamental mechanisms underlying coordinated RNA processing events that contribute to gene expression may provide new insights into its dysfunction in disease and the potential for the development of biomarkers and therapeutic strategies.

### Limitations of the study

The EIF4A3 SMASh tag yielded only ∼66% protein depletion in 24 h, meaning that effects on target genes were likely milder than a strong depletion or true knockout. However, it is well established in the RNA processing field that even twofold changes in protein concentration are sufficient to produce major switches in splicing (Zuo and Manley 1993; Ellis et al. 2023). Compared with short-read sequencing, which yielded ∼40 million reads per replicate, our long-read sequencing data sets contained ∼500,000 reads per replicate. Hence, the long-read data sets cannot provide the same gene coverage for lowly expressed genes. Future advancements in long-read sequencing techniques will provide greater depth of sequencing and expand the number of genes that can provide insights into the mechanisms of cotranscriptional splicing regulation.

## Materials and methods

### Cell culture and generation of stable cell lines

SMASh-EIF4A3 HEK293T cells were generated as described previously by Park et al. (2014), by exchanging the DD tag for a SMASh tag (pCS6-SMASh-YFP, plasmid 68852). HEK293T cells, SMASh-EIF4A3-expressing HEK293T cells, Flp-In T-REx 293 cells (Thermo Fisher Scientific R78007), and Flp-In T-REx 293–EIF4A3-overexpressing cells were cultivated at 37°C in Dulbecco's modified Eagle's medium (DMEM) GlutaMAX (Thermo Fisher Scientific 10569010) supplemented with 10% fetal bovine serum (Gibco A5670701) and 1% penicillin/streptomycin (Thermo Fisher Scientific 15140122). Medium for SMASh-EIF4A3 cells was supplemented with 1 μg/mL puromycin (Thermo Fisher Scientific A1113803) and 10 μg/mL blasticidin (Thermo Fisher Scientific A1113903). Medium for Flp-In T-REx 293 wild-type cells was supplemented with 100 μg/mL zeocin (Thermo Fisher Scientific R25001) and 15 μg/mL blasticidin, and medium for Flp-In T-REx 293–EIF4A3 cells was supplemented with 15 μg/mL blasticidin and 150 μg/mL hygromycin (Thermo Fisher Scientific 10687010).

### Generation of EIF4A3-overexpressing Flp-In T-REx 293 cells

An EIF4A3 gBlock (IDT) was PCR-amplified with BamHI and XhoI overhangs (Supplemental Table S2) using PrimeSTAR Max DNA polymerase (Takara R045A). PCR product was digested with BamHI-HF (NEB R3136S) and XhoI (NEB R0146S), purified with QIAquick PCR and gel cleanup kit (Qiagen 28506), ligated with pCDNA5-FRT/TO (Thermo Fisher Scientific V6520-20), digested, and gel-purified with the same restriction enzymes and cleanup kit using T4 DNA ligase (NEB M0202L). Correct insertion of the transgene was confirmed via Sanger sequencing (Quintara Bio). For the generation of a stable cell line, 800,000 Flp-In T-REx 293 cells in antibiotic-free medium were cotransfected with the EIF4A3-pCDNA5 vector and pOG44 (Thermo Fisher Scientific V600520) in a 1:9 DNA concentration ratio using Lipofectamine 2000 (Thermo Fisher Scientific 11668027) in a 6 well plate. Twenty-four hours after transfection, cells were replated in different dilutions, and 48 h after transfection, medium was changed to selection medium (medium supplemented with 15 μg/mL blasticidin and 150 μg/mL hygromycin). Selection medium was changed every 3 days. EIF4A3 expression was induced with 0.1 μg/mL doxycycline for 48 h.

### Rescue experiment

SMASh-EIF4A3 HEK293T cells were seeded at 144,000 cells per well in 6 well plates 24 h prior to transfection with 2 μg/well EIF4A3-pCDNA5 vector. Transfection was carried out using Lipofectamine 3000 (Thermo Fisher Scientific L3000015) following the manufacturer's instructions. Twenty-four hours after transfection, depletion and rescue cells were treated with 1.5 μM danoprevir. All cells were harvested in 500 μL of ice-cold PBS 24 h after danoprevir treatment.

### Subcellular fractionation

Three million SMASh-EIF4A3 HEK93T cells or HEK293T wild-type cells were seeded in 30 mL of medium on 150 mm dishes (four dishes per biological replicate) 24 h prior to treatment with 1.5 μM danoprevir or the same volume of DMSO. Fractionation was carried out 24 h after treatment as described previously by Reimer and Neugebauer (2020), with the following adjustments: Cells were harvested in 5 mL of ice-cold PBS using a cell scraper and transferred to 15 mL Falcon tubes. Each cell dish was treated as an individual sample, and samples corresponding to the same biological replicate were pooled after nascent RNA extraction. Cells were centrifuged at 240*g* for 7 min at 4°C and then processed according to the protocol starting at step 2. Washing of nuclear and chromatin pellets (steps 9 and 15) was repeated twice for a total of three washes, respectively.

### Western blot

Cytoplasm, nucleoplasm, and chromatin fractions from cell fractionation were adjusted to equal volumes with PBS. Nucleoplasm and chromatin fractions were homogenized by sonication, and all samples were spun at 14,000 rpm for 10 min at 4°C before gel loading. Western blots were performed with antibodies against Pol II 4H8 (Santa Cruz Biotechnology sc-47701) and tubulin (Abcam ab6161) at 1:1000 dilution overnight at 4°C. To test EIF4A3 depletion or overexpression, antibodies against EIF4A3 (Proteintech 677401) and GAPDH (Abcam 9485) were used at 1:1000 overnight at 4°C.

### Total RNA extraction

For EIF4A3 depletion and danoprevir treatment control, 125,000 SMASh-EIF4A3 HEK293T cells or HEK293T wild-type cells were seeded in 6 well plates 24 h prior to treatment. Subsequently, cells were treated for 24 h with either 1.5 μM danoprevir (Selleckchem S1183) dissolved in DMSO or the same volume of DMSO. For EIF4A3 overexpression, 400,000 Flp-In T-REx 293-EIF4A3 cells were seeded in 6 wells 24 h prior to expression induction with 0.1 μg/mL doxycycline (control cells were untreated) for 48 h. To harvest, cells were washed off the wells with 500 μL of ice-cold PBS and then centrifuged at 1000*g* for 1 min. PBS was removed, cells were resuspended in 1 mL of TRIzol (Thermo Fisher Scientific 15596018), and RNA was extracted according to the manufacturer's protocol. Subsequently, RNA was further purified via the RNeasy mini kit (Qiagen 74104), including DNase digestion (RNase-free DNase set, Qiagen 79254).

### Nascent RNA (nRNA) extraction

Nascent RNA was extracted from chromatin as described previously (Reimer and Neugebauer 2020). For Illumina short-read sequencing, the protocol was followed until step 40 [including poly(A)^+^ depletion but excluding ribosomal RNA (rRNA) depletion], and samples were subsequently used for library preparation, which included rRNA depletion (see below).

### Library preparation for long-read sequencing of mRNA on the Pacific Biosciences platform

RNA quantity and quality were assessed using on-chip capillary electrophoresis on a Bioanalyzer 2100 (Agilent Technologies). The Iso-Seq libraries were prepared according to Pacific Bioscience's “Preparing Iso-Seq Libraries using SMRTbell” preparation kit 3.0 manual and loaded onto the Pacific Biosciences Sequel II sequencer. See the Supplemental Material for further details.

### Library preparation for Illumina paired-end sequencing of poly(A)^+^ RNA

For RNA-seq quality control, total RNA quality was determined by estimating the A260/A280 and A260/A230 ratios by nanodrop. RNA integrity was determined by running an Agilent Bioanalyzer gel, which measured the ratio of the ribosomal peaks. For details about library preparation, see the Supplemental Material.

### Library preparation for Illumina paired-end sequencing of nascent RNA (nRNA)

RNA-seq quality control was performed as above [see “Library Preparation for Illumina Paired-End Sequencing of Poly(A)^+^ RNA”]. rRNA was depleted using the KAPA RNA HyperPrep kit with RiboErase (KR1351). Flow cell preparation and sequencing were as for mRNA. See the Supplemental Material for further details.

### RT-PCR for validation experiments

Reverse transcription was carried out using 500 ng of total RNA, random hexamer (Thermo Fisher Scientific SO142), and SuperScript III reverse transcriptase (Thermo Fisher Scientific 18080093) according to the manufacturer's instructions. cDNA was amplified with PrimeSTAR Max DNA polymerase (Takara R047A) and gene-specific primers (Supplemental Table S2). Amplification products were visualized via 1% agarose gel electrophoresis.

### Illumina sequencing of mRNA data analysis

Paired-end read mates were generated in .fastq format. Illumina adapters were trimmed using fastp 0.24.0 (Chen et al. 2018). Reads were mapped to the GRCh38.p14 genome (GENCODE release 44) using STAR 2.7.11b (Dobin et al. 2013) with settings ‐‐runMode alignReads -outSAMtype BAM SortedByCoordinate. The resulting .bam files were indexed with SAMtools 1.21 (Danecek et al. 2021). Splicing was quantified by running Whippet (Sterne-Weiler et al. 2018) on fastp outputs. Gene expression was determined using featureCounts (subread 2.0.8) (Liao et al. 2014) followed by DESeq2 (Love et al. 2014). All downstream analyses were carried out using custom Python scripts that use the output files from DESeq2 and Whippet.

### Data analysis of publicly available data sets

Illumina short-read data sets of siRNA knockdown against EJC components from Wang et al. (2014) with GEO accession number GSE63091 and Rodor et al. (2016) with GEO accession number GSE81460 were downloaded and processed in the same way as our own mRNA Illumina short-read data set (described above). Data processed from the ENCODE consortium are listed in Supplemental Table S3.

### Illumina sequencing of nRNA data analysis

Paired-end read mates were generated in .fastq format. Illumina adapters were trimmed using fastp 0.24.0 (Chen et al. 2018). Reads were mapped to the GRCh38.p14 genome (GENCODE release 44) using STAR 2.7.11b (Dobin et al. 2013) with settings ‐‐runMode alignReads –outSAMattributes All ‐‐outSAMtype BAM ‐‐SortedByCoordinate. The resulting .bam files were indexed with SAMtools 1.21 (Danecek et al. 2021). Junction reads were quantified using splice-q 1.0.0 (de Melo Costa et al. 2021), and splicing per intron (SPI) values were calculated using a custom Python script using the SPLICE-q output files. Splicing order was calculated using Insplico (Gohr et al. 2023) with the STAR outputs as input.

### Analysis of long-read sequencing of mRNA data

Adapters were trimmed, and data were preprocessed using fastplong 0.2.2 (Chen 2023). Reads were mapped to the GRCh38.p14 genome (GENCODE release 44) using FLAIR align 2.0.0 (Tang et al. 2020). Block-skipping junction reads were identified using a custom Python script that identifies deletions in reads based on the CIGAR string in the .bam output files of FLAIR align.

### Analysis of alternative splicing modules with MAJIQ

ENCODE RBP knockdown short-read poly(A)-selected RNA-seq data were analyzed with MAJIQ using batch correction with MOCCASIN, as described previously (Slaff et al. 2021; Vaquero-Garcia et al. 2023). Briefly, batch correction was performed using MOCCASIN on K562 or HepG2 data separately. Knockdowns for each RBP in each cell type were quantified by comparing the duplicate depletion experiment versus a composite control group of all duplicate controls from all batches in the matching cell type using MAJIQ HET. Significant changes upon knockdown were the junctions or introns with an absolute median difference in expected PSI of ≥20% and a Mann–Whitney two-sided *P* < 0.05. The VOILA Modulizer was run on the resulting outputs to define alternative splicing modules and alternative splicing event types by using the option decomplexify-psi-threshold 0.05. This removed all junctions and introns from the splice graph that had a median E(PSI) of <5% across all experimental groups. Block exons were defined as events that were categorized under the “tandem cassette exon” and/or “multiexon-spanning” event subtypes that remained after removing low inclusion junctions. Block exons were only considered to be regulated by an RBP if the block exon had a significant block-skipping junction and significant changing junctions between both flanking constitutive exons and the first and last exon of the block using the above changing definitions. The same thresholds were applied to all the junctions of other event types (cassette exon, alt 3′ss, alt 5′ss, intron retention, alternative first and last exon, etc.) for an event to be considered regulated by that RBP. We focused on RBPs that had the highest fraction of regulated events being the block exon type and plotted the relative enrichment of block exon regulation for each RBP compared with the median rate of block exon regulation across all RBPs that had at least one regulated block exon (see Supplemental Table S3).

We assessed the similarity between our EIF4A3 depletion results in HEK293T cells and EJC component depletions from other cell types from ENCODE HepG2 and K562 cells and from HeLa cells (Wang et al. 2014) as follows: All depletion experiments were quantified with MAJIQ, and we determined which AS events (MAJIQ local splicing variations) were coregulated in every pairwise comparison (absolute dPSI of ≥20%). Among coregulated events in every pair, we calculated Pearson correlation coefficients for dPSI values and the significance of the number of coregulated events using a two-sided Fisher's exact test. These results are summarized in Supplemental Table S4.

### Resource availability

Requests for further information and materials should be addressed to the lead contact, K.M.N. (karla.neugebauer@yale.edu).

### Data and code availability

Raw and processed RNA long- and short-read sequencing data generated in this study have been deposited in NCBI's Gene Expression Omnibus and are accessible through GEO series accession number GSE291593. All codes used for data analysis and for generating figures in this manuscript are available at https://github.com/NeugebauerLab/exon-blocks. Illumina short-read data sets of siRNA knockdown against EJC components from Wang et al. (2014) analyzed in this manuscript is available at GEO with accession number GSE63091. The Acinus knockdown is accessible under accession number GSE81460.

## Supplemental Material

Supplement 1

Supplement 2

Supplement 3
