# Supplementary material for: The exon junction complex coordinates the cotranscriptional inclusion of blocks of neighboring exons
Source: Genes Dev. 2026 Jan 1;40(1-2):94–109. doi: 10.1101/gad.353081.125 (PMC12758388; doi:10.1101/gad.353081.125)
Supplement: Supplement 3 [file Supplemental.docx]

**SUPPLEMENTAL MATERIAL**

**The Exon Junction Complex coordinates**

**the co-transcriptional inclusion of blocks of neighboring exons**

Alexandra Bergfort^1^, Jackson M. Gordon^1^, Matthew Gazzara^2,3^, Chuan-Tien Hung^3^, Benhur Lee^3^, Yoseph Barash^2,3^, and Karla M. Neugebauer^1^

^1^Department of Molecular Biophysics and Biochemistry, Yale University, New Haven CT 06511, USA.

^2^Department of Genetics, University of Pennsylvania, Philadelphia, PA 19104, USA.

^3^Department of Computer and Information Science, University of Pennsylvania, Philadelphia, PA, USA.

^4^Department of Microbiology, Icahn School of Medicine at Mount Sinai, New York, NY 10029, USA.

**Contents of Supplemental material**

**Supplemental Figures:**

**Supplemental Figure S1:** Related to Figure 1 and 2. Danoprevir treatment control, permutation tests for altered splice events per gene and overlap of altered splice events upon EIF4A3 depletion in HEK293T and HeLa cells.

**Supplemental Figure S2:** Related to Figure 3. RT-PCR validations, rescue experiment and exon classification.

**Supplemental Figure S3:** Splicing changes observed upon EIF4A3 depletion are unlikely due to weakened NMD. Related to Figure 3.

**Supplemental Figure S4:** Related to Figure 5. Overlap of mRNA and nRNA altered genes and splicing order surrounding block exons.

**Supplemental Figure S5:** Related to Figure 6. Logo plots of block exon splice sites, block exon gene length and block exon length.

**Supplemental Figure S6:** GO term analysis of genes affected by EIF4A3 depletion.

**Supplemental Figure S7:** Recursive splice site strength analysis of single EIF4A3 regulated exons and block exons.

**Supplemental Methods**

**Supplemental Tables:**

**Supplemental Table S1:** Mapping statistics of RNAseq data sets analyzed in this study (included in this pdf).

**Supplemental Table S2:** Oligonucleotides used in this study (included in this pdf).

**Supplemental Table S3:** Results of splicing analysis of RBP knockdowns from ENCODE consortium (xlsx file).

**Supplemental Table S4:** overlap of regulated splice events between data sets and correlation of dPSI values of overlapping events (xlsx file).

**
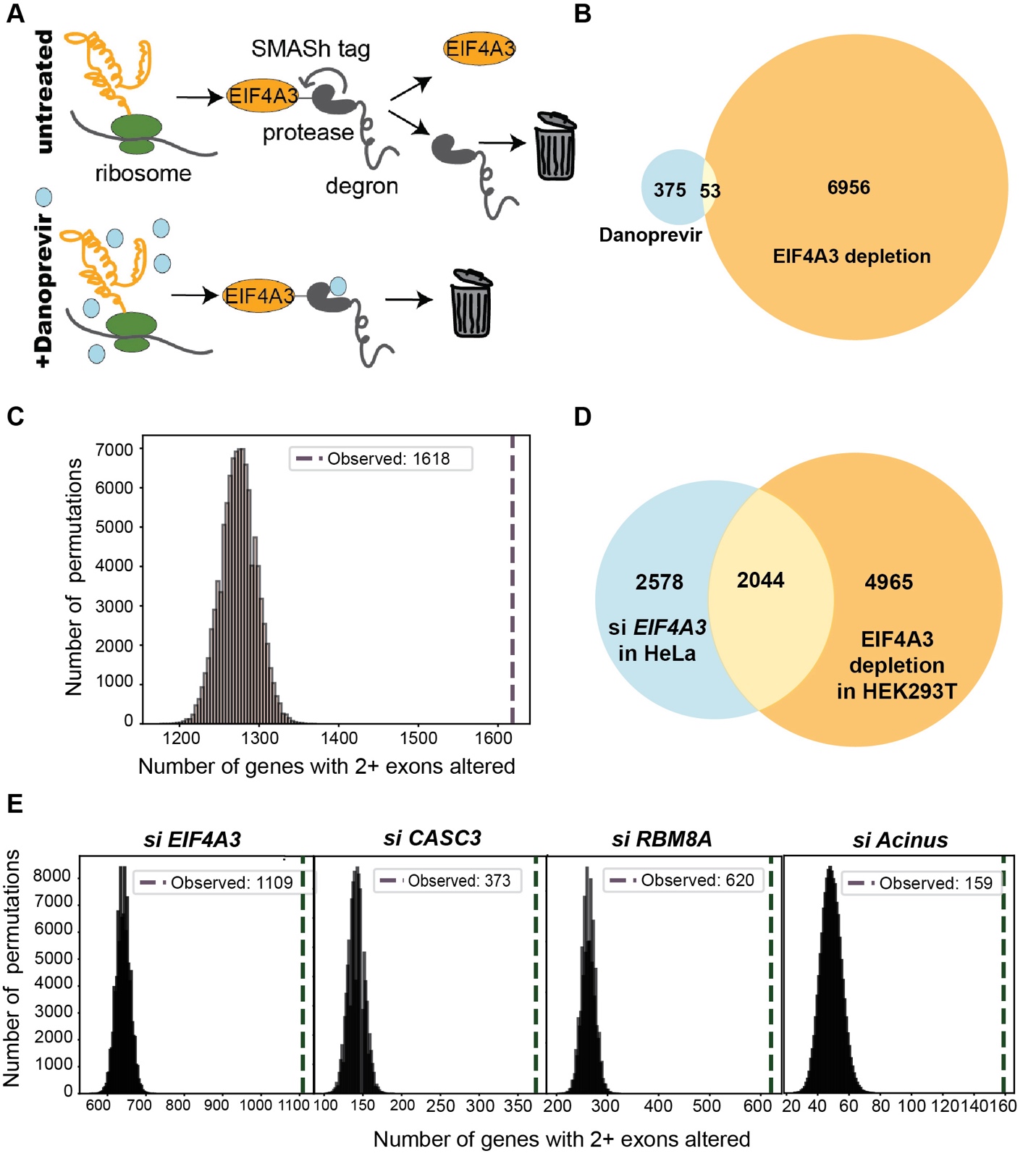
**

**Supplemental Figure S1: Related to Figure 1 and 2. (A)** Schematic of the SMASh degron method. Adapted from Chung et al. 2015. **(B)** Overlap of cassette exons differentially spliced in HEK293T cells treated with Danoprevir (light blue) and SMASh-EIF4A3 depleted HEK293 cells (orange). (**C)** Permutation test (100,000 iterations) evaluating the likelihood of ≥2 differentially spliced exons per gene in EIF4A3-depleted HEK293T cells, based on expressed genes and observed splicing changes. (**D)** Overlap of cassette exons differentially spliced in HEK293T cells and HeLa cells upon EIF4A3 depletion. (**E**) Like **(**B) but with previously published EJC siRNA knockdown in HeLa cells. Data sets accessible under GEO database GSE63091 and GSE81460.


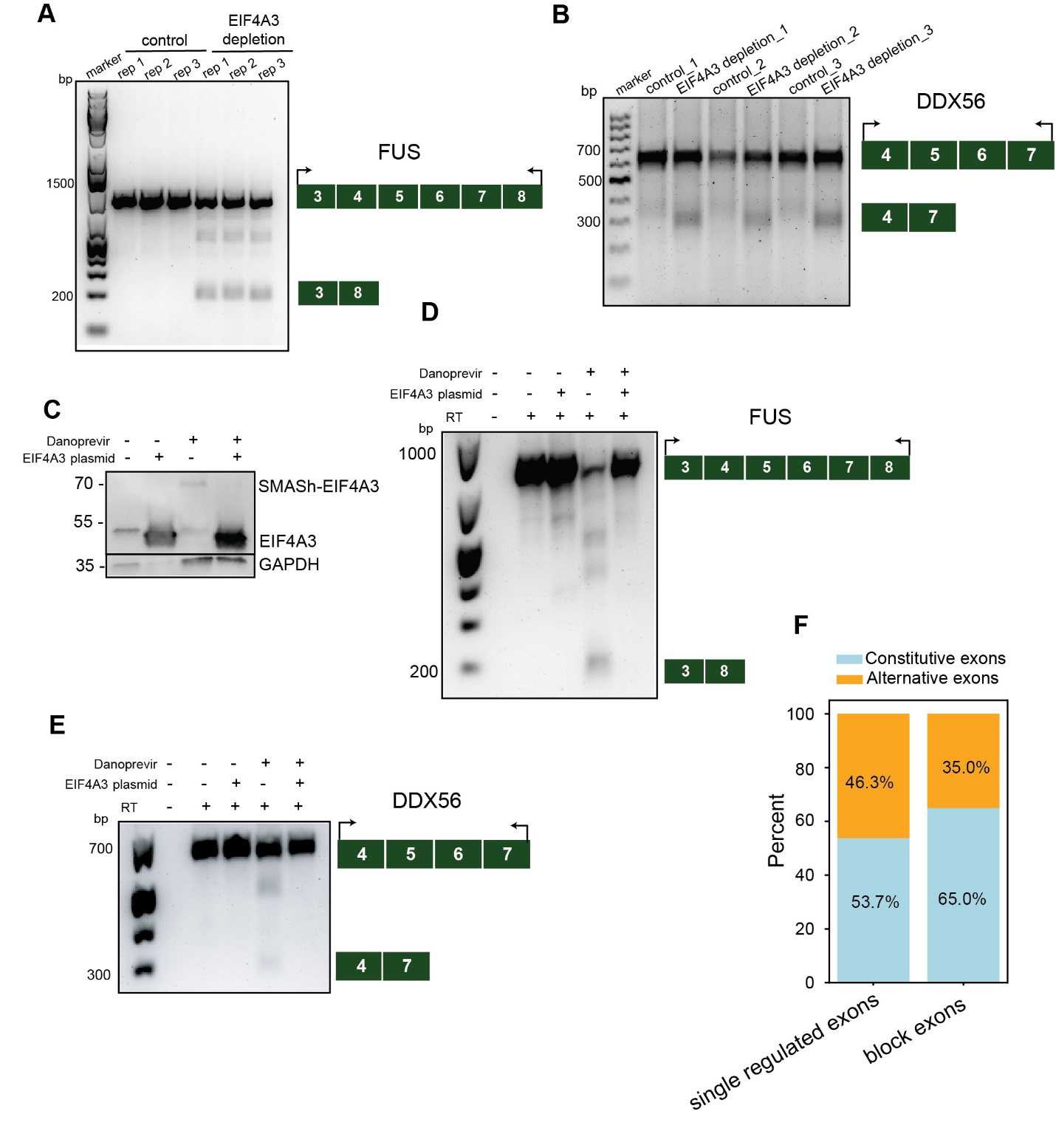


**Supplemental Figure S2: Related to Figure 3. (A)** Agarose gel showing RT-PCR on total RNA extracted from control and EIF4A3 depletion cells with primer pairs targeting *FUS* exon 3 and 8. Expected products are shown to the right. Molecular weight is indicated in base pairs (bp) to the left. (**B)** Like (A) but with primers targeting *DDX56* exon 4 and 7. **(C)** Western blot showing EIF4A3 expression levels in rescue experiment. **(D)** and **(E)** Agarose gels with RT-PCR products for *FUS* exon 3-8 and *DDX56* exon 4-7 in samples of rescue experiment. **(F)** Stacked bar chart illustrating percentages of EIF4A3 regulated exons classified as constitutive (present in all hg38 annotated isoforms, regardless of cell line) or alternative exons.


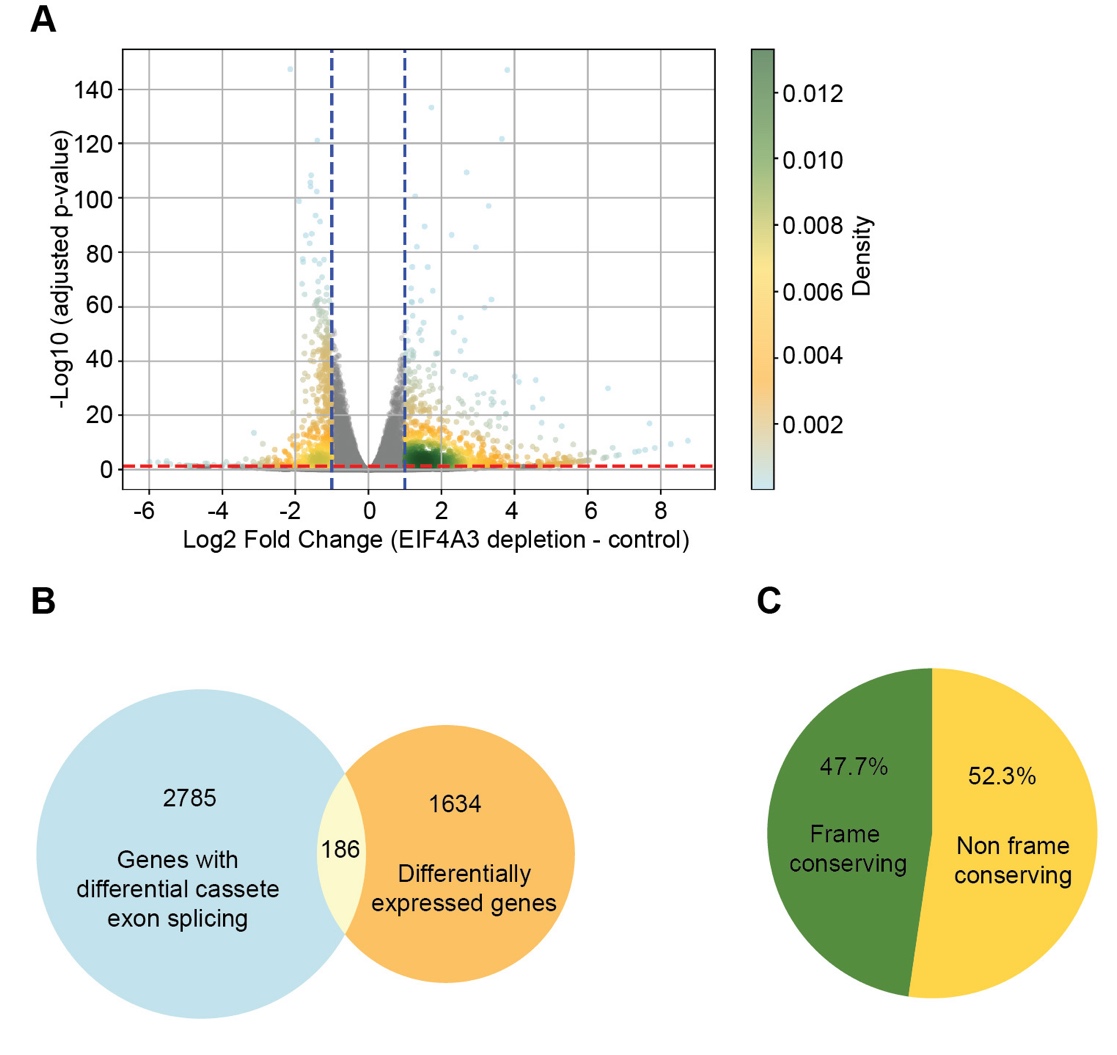


**Supplemental Figure S3: Splicing changes observed upon EIF4A3 depletion are unlikely due to weakened NMD. (A)** Gene expression analysis with log2 Fold Change (x-axis) versus adjusted p-value (y-axis). Threshold: log2 Fold Change of 1/-1 and p-value ≥ 0.05 (indicated by blue and red dotted lines); color reflects local point density (2D Gaussian KDE). **(B)** Venn diagram showing overlap of genes with splicing alterations and genes with altered gene expression upon EIF4A3 depletion. **(C)** Pie chart illustrating percentage of exon block skipping events that is frame conserving (green) versus non-frame conserving (yellow).


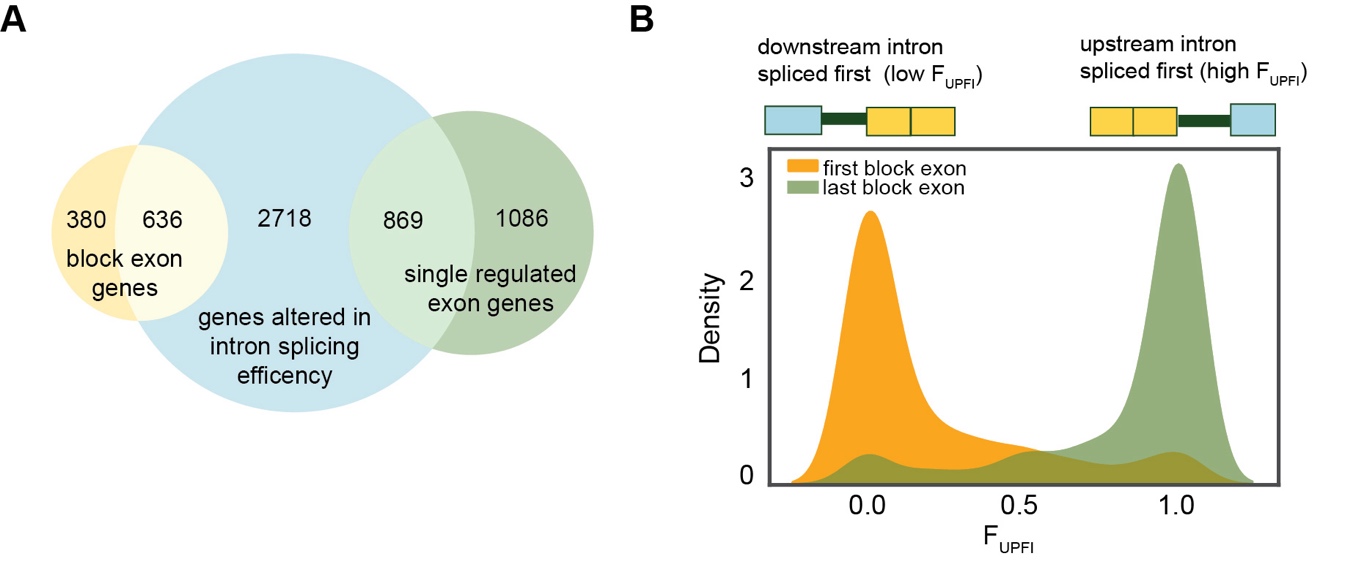


**Supplemental Figure S4: Related to Figure 5. (A)** Venn diagram, illustrating overlap of genes with block exons, single EIF4A3 regulated exons and genes with introns altered in splicing efficiency upon EIF4A3 depletion. (**B)** Splicing order of introns surrounding the first (orange) and last (green) exon of coordinated exon blocks in EIF4A3 depletion cells. Y-axis: Probability density (Kernel Density Estimate), x-axis: fraction of upstream intron spliced first (F_UPFI_).


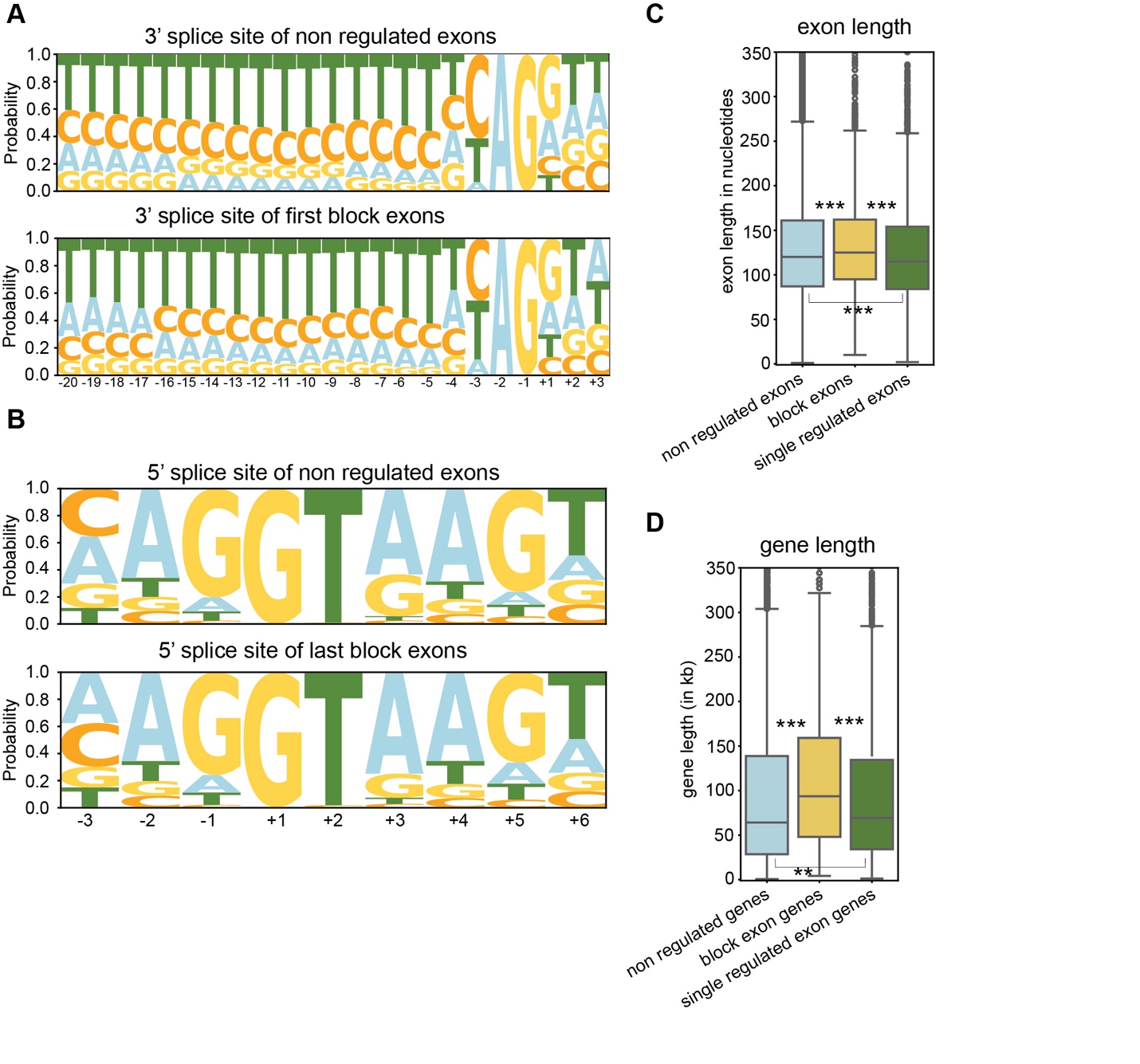


**Supplemental Figure S5: Related to Figure 6.** (**A**) Logo-plots of 3’splice sites of non EIF4A3 regulated exons versus first block exons. Nucleotide positions are indicated below the bottom logo. **(B)** like (A) but for 5’ splice site strength. **(C)** Box plot showing exon length of non-EIF4A3 regulated exons (light blue), block exons (yellow) and single regulated exons (green). Significance is indicated by asterisk (***: p<0.001, **: p< 0.01). (**D)** Like (C) but for gene length.

**
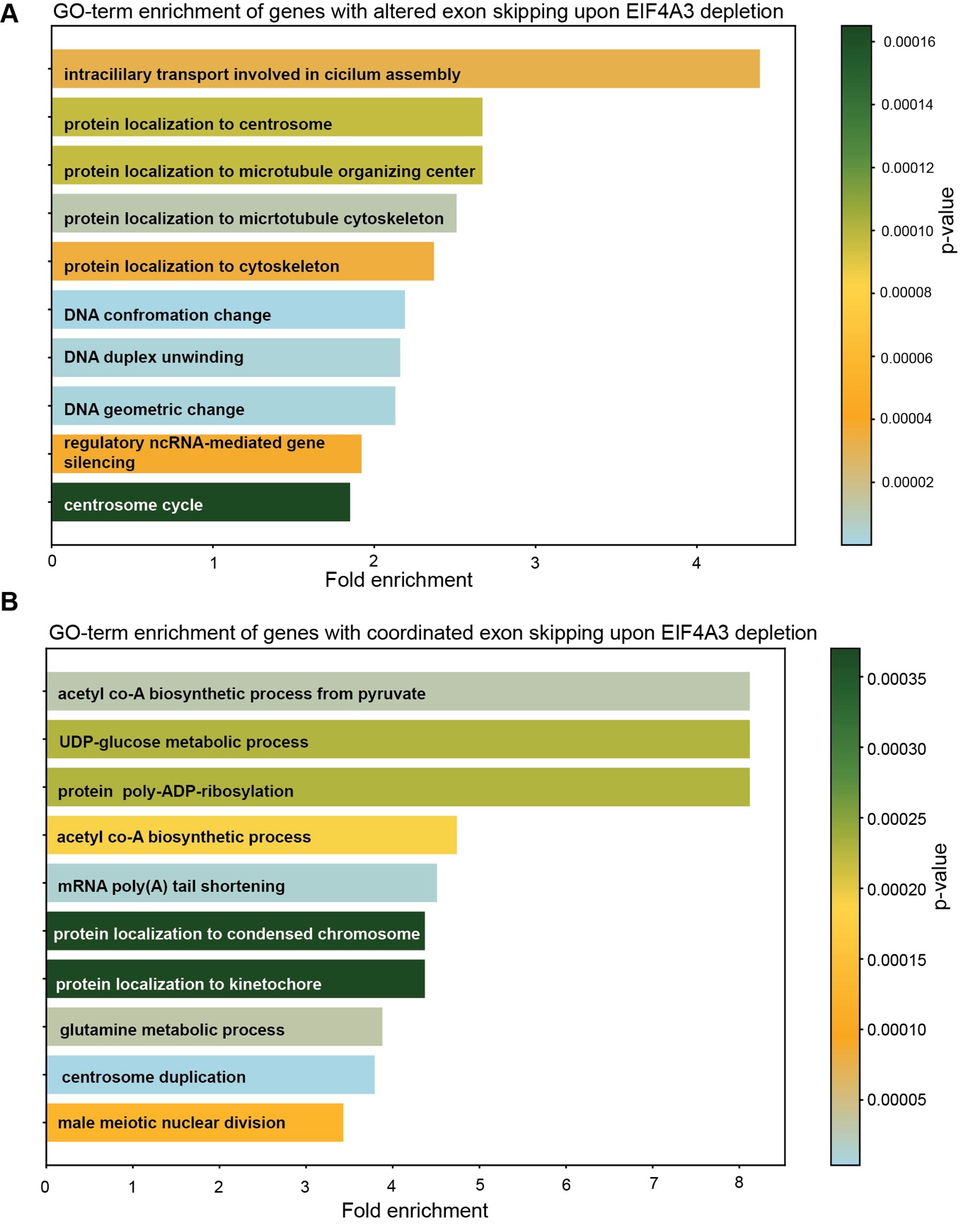
**

**Supplemental Figure S6: EIF4A3 splicing regulated genes are slightly enriched in cell division associated GO-terms. (A)** GO-term analysis “biological process” of all genes with altered exon splicing upon EIF4A3 depletion. Fold enrichment on the x-axis. P-value is color coded according to the bar to the right. **(B)** Same as in (A) but for genes with coordinated exon skipping upon EIF4A3 depletion.


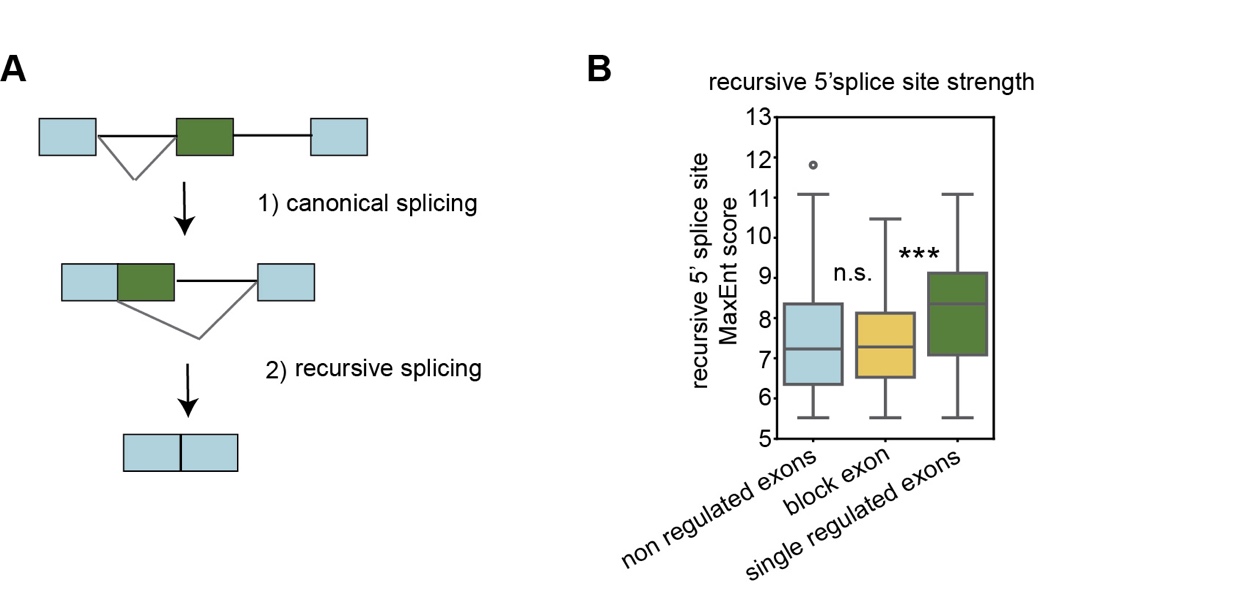


**Supplemental Figure S7: Recursive splicing is not a mechanism underlying block exon skipping. (A)** Schematic of the process of recursive splicing. In the first step a canonical splicing reaction takes place. In the second step, the newly formed exon-exon junction is misinterpreted as a 5’ splice site (recursive 5’ splice site), leading to excision of the middle exon. (**B)** Box plot with MaxEnt scores for recursive 5’ splice sites of non-EIF4A3 regulated exons (light blue), block exons (yellow) and single EIF4A3 regulated exons (green). Significance is indicated by asterisk (***: p<0.001, n.s.: not significant).

**Supplemental Methods**

**Library preparation for long-read sequencing of mRNA on the Pacific Biosciences platform**

The Iso-Seq libraries were prepared according to Pacific Bioscience’s Preparing Iso-Seq® Libraries using SMRTbell® Prep Kit 3.0 manual. Briefly, 300 ng of RNA was used for reverse transcription and template switching. cDNAs for each sample were generated using the recommended barcoded NEBNext single-cell cDNA PCR primers and Iso-Seq Express cDNA PCR primers listed in Appendix 3 of the manual. The cDNAs from the 6 samples were pooled equimolarly, 1.05x SMRTbell clean-up was done to size select 500bp and above fragments and one SMRT library was prepared and run on one SMRTcell. Primer annealing and polymerase binding reactions were prepared using the Binding Calculator from Pacific Biosciences, based on sample volume, concentration, and insert size using default settings. The SMRTbell libraries were annealed to sequencing primers and then bound to polymerase using the Sequel® II Binding Kit 3.1 before being loaded onto the Pacific Biosciences Sequel II sequencer. Sequencing was performed for 24 hours of movie time.

**Library preparation for Illumina paired end sequencing of poly(A)+-RNA**

mRNA was purified from approximately 200ng of total RNA with oligo-dT beads and sheared by incubation at 94C in the presence of Mg (Roche Kapa mRNA Hyper Prep Cat# KR1352). Following first-strand synthesis with random primers, second strand synthesis and A-tailing were performed with dUTP for generating strand-specific sequencing libraries. Adapter ligation with 3’ dTMP overhangs were ligated to library insert fragments. Library amplification amplifies fragments carrying the appropriate adapter sequences at both ends. Strands marked with dUTP were not amplified. Indexed libraries were quantified by qRT-PCR using a commercially available kit (Roch KAPA Biosystems Cat# KK4854) and insert size distribution determined by the Agilent Bioanalyzer. Samples with a yield of ≥ 0.5 ng/ul and a size distribution of 150-300bp were used for sequencing.

Sample concentrations were normalized to 2nM and loaded onto an Illumina NovaSeq X Plus flow cell at a concentration that yields 25 million passing filter clusters per sample. Samples were sequenced using 101bp paired-end sequencing on an Illumina NovaSeq X Plus according to Illumina protocols. The 10bp unique dual index was read during additional sequencing reads that automatically follow the completion of read 1. Data generated during sequencing runs were simultaneously transferred to the Yale Center for Genome Analysis (YCGA) high-performance computing cluster. A positive control (prepared bacteriophage ΦX174 library) provided by Illumina was spiked into every lane at a concentration of 0.3% to monitor sequencing quality in real time.

**Library preparation for Illumina paired end sequencing of nascent RNA (nRNA)**

Using the Kapa RNA HyperPrep Kit with RiboErase (KR1351), rRNA was depleted starting from 500 ng of total RNA by hybridization of rRNA to complementary DNA oligonucleotides, followed by treatment with RNase H and DNase to remove rRNA duplexed to DNA. Samples were then fragmented using heat and magnesium. 1^st^ strand synthesis was performed using random priming. 2^nd^ strand synthesis incorporated dUTPs into the 2^nd^ strand cDNA. Adapters were then ligated, and the library was amplified. Strands marked with dUTPs were not amplified allowing for strand-specific sequencing. Indexed libraries that met appropriate cut-offs for both quantity and quality were quantified by qRT-PCR using a commercially available kit (KAPA Biosystems) and insert size distribution determined with Agilent Bioanalyzer. Samples with a yield of ≥0.5 ng/ul were used for sequencing.

Flow Cell Preparation and Sequencing: as above for mRNA.

**GO term analysis**

To identify enriched biological processes, we performed GO term analysis using the Gene Ontology server (geneontology.org). The list of genes of interest was uploaded, specifying Homo sapiens as the reference genome. We focused on biological processes as the category of interest. After obtaining the results, we exported the data table and processed it using a custom Python script.

| **Sample** | **Replicate** | **Platform** | **Uniquely mapped reads** | **Mean Read length** |
| --- | --- | --- | --- | --- |
| smash_DMSO_mRNA | 1 | Illumina NovaSeq X Plus | 44006193 | 265.76 |
| smash_DMSO_mRNA | 2 | Illumina NovaSeq X Plus | 47978920 | 263.2 |
| smash_DMSO_mRNA | 3 | Illumina NovaSeq X Plus | 42550733 | 261.62 |
| smash_Danoprevir_mRNA | 1 | Illumina NovaSeq X Plus | 53190910 | 263.44 |
| smash_Danoprevir_mRNA | 2 | Illumina NovaSeq X Plus | 48627782 | 259.36 |
| smash_Danoprevir_mRNA | 3 | Illumina NovaSeq X Plus | 56253278 | 260.73 |
| smash_DMSO_nRNA | 1 | Illumina NovaSeq X Plus | 105457358 | 285.22 |
| smash_DMSO_nRNA | 2 | Illumina NovaSeq X Plus | 106338256 | 286.92 |
| smash_DMSO_nRNA | 3 | Illumina NovaSeq X Plus | 75203790 | 289.52 |
| smash_Danoprevir_nRNA | 1 | Illumina NovaSeq X Plus | 81410858 | 288.92 |
| smash_Danoprevir_nRNA | 2 | Illumina NovaSeq X Plus | 85707025 | 286.34 |
| smash_Danoprevir_nRNA | 3 | Illumina NovaSeq X Plus | 93254009 | 285.52 |
| smash_DMSO_mRNA | 1 | PacBio Sequel II | 563502 | 1731.6 |
| smash_DMSO_mRNA | 2 | PacBio Sequel II | 948289 | 1712.3 |
| smash_DMSO_mRNA | 3 | PacBio Sequel II | 651560 | 1725.36 |
| smash_Danoprevir_mRNA | 1 | PacBio Sequel II | 820105 | 1688.68 |
| smash_Danoprevir_mRNA | 2 | PacBio Sequel II | 645916 | 1714.06 |
| smash_Danoprevir_mRNA | 3 | PacBio Sequel II | 562241 | 1681.43 |
| EIF4A3_OE_ctr_mRNA | 1 | Illumina NovaSeq X Plus | 24097972 | 263.48 |
| EIF4A3_OE_ctr_mRNA | 2 | Illumina NovaSeq X Plus | 43767393 | 261.15 |
| EIF4A3_OE_ctr_mRNA | 3 | Illumina NovaSeq X Plus | 29986736 | 263.81 |
| EIF4A3_OE_mRNA | 1 | Illumina NovaSeq X Plus | 37923570 | 262.2 |
| EIF4A3_OE_mRNA | 2 | Illumina NovaSeq X Plus | 40263385 | 259.79 |
| EIF4A3_OE_mRNA | 3 | Illumina NovaSeq X Plus | 34385463 | 271.5 |
| HEK293T_DMSO_mRNA | 1 | Illumina NovaSeq X Plus | 21903428 | 274.83 |
| HEK293T_DMSO_mRNA | 2 | Illumina NovaSeq X Plus | 29153066 | 272.54 |
| HEK293T_DMSO_mRNA | 3 | Illumina NovaSeq X Plus | 28170338 | 273.61 |
| HEK293T_Danoprevir_mRNA | 1 | Illumina NovaSeq X Plus | 29744457 | 273.68 |
| HEK293T_Danoprevir_mRNA | 2 | Illumina NovaSeq X Plus | 31377966 | 272.99 |
| HEK293T_Danoprevir_mRNA | 3 | Illumina NovaSeq X Plus | 28170338 | 273.61 |

**Supplemental Table S1: Mapping statistics of RNAseq data sets analyzed in this study.**

**Supplemental Table S2: Oligonucleotides used in this study**

| **Description** | **Sequence** |
| --- | --- |
| BamHI_eif4a3_F | TAAGCAGGATCCATGGCGACCACGGCCACGAT |
| XhoI_eiF4a3_R | TAAGCACTCGAGTCAGATAAGATCAGCAACGTTCATCGGC |
| FUS_exon3_F | CTATGGGGCCTACCCCACCC |
| FUS_exon8_R | CGGAGTCATGACGTGATCCTTGGTC |
| DDX56_exon4_F | AGCTGTGCTGATGGAGAAGCC |
| DDX56_exon7_R | TTGTCCCCTTTGGGCCCTC |
